# The Stress Granule RNA-Binding Protein TIAR-1 Protects Female Germ Cells from Heat Shock in *Caenorhabditis elegans*

**DOI:** 10.1534/g3.115.026815

**Published:** 2016-02-09

**Authors:** Gabriela Huelgas-Morales, Carlos Giovanni Silva-García, Laura S. Salinas, David Greenstein, Rosa E. Navarro

**Affiliations:** * Departamento de Biología Celular y Desarrollo, Instituto de Fisiología Celular, Universidad Nacional Autónoma de México, Ciudad de México 04510, Mexico.; † Department of Genetics, Cell Biology and Development, University of Minnesota Minneapolis, 55455 Minnesota

**Keywords:** stress granules, TIA-1/TIAR, germ cells, stress, *C. elegans*

## Abstract

In response to stressful conditions, eukaryotic cells launch an arsenal of regulatory programs to protect the proteome. One major protective response involves the arrest of protein translation and the formation of stress granules, cytoplasmic ribonucleoprotein complexes containing the conserved RNA-binding proteins TIA-1 and TIAR. The stress granule response is thought to preserve mRNA for translation when conditions improve. For cells of the germline—the immortal cell lineage required for sexual reproduction—protection from stress is critically important for perpetuation of the species, yet how stress granule regulatory mechanisms are deployed in animal reproduction is incompletely understood. Here, we show that the stress granule protein TIAR-1 protects the *Caenorhabditis elegans* germline from the adverse effects of heat shock. Animals containing strong loss-of-function mutations in *tiar-1* exhibit significantly reduced fertility compared to the wild type following heat shock. Analysis of a heat-shock protein promoter indicates that *tiar-1* mutants display an impaired heat-shock response. We observed that TIAR-1 was associated with granules in the gonad core and oocytes during several stressful conditions. Both gonad core and oocyte granules are dynamic structures that depend on translation; protein synthesis inhibitors altered their formation. Nonetheless, *tiar-1* was required for the formation of gonad core granules only. Interestingly, the gonad core granules did not seem to be needed for the germ cells to develop viable embryos after heat shock. This suggests that TIAR-1 is able to protect the germline from heat stress independently of these structures.

When exposed to stress, cells undertake a series of adaptive responses to ensure survival. Among these responses are the reprogramming of gene expression, which involves a global arrest in translation, and the formation of ribonucleoprotein complexes (RNPs). In stressful conditions, total protein synthesis decreases, but proteins needed to contend with stress continue to be translated (Arribere *et al.* 2011). Among the RNPs that form under stress are stress granules and processing bodies (P bodies) (Kedersha *et al.* 2005; Wilczynska *et al.* 2005). P bodies are thought to constitute sites of messenger RNA (mRNA) degradation, as they contain components of the 5′-to-3′ mRNA decay machinery. Although P bodies are detected in normal conditions, they increase in size and number under stressful conditions (Wilczynska *et al.* 2005). Stress granules contain stalled translation initiation complexes, and have been proposed as sites where untranslated mRNAs are temporally stored (reviewed in Anderson and Kedersha 2008). After normal conditions are restored, stress granules dissociate, and mRNA translation continues (Kedersha *et al.* 2000).

The T-cell-restricted intracellular antigen proteins (TIA-1/TIAR) are central players in stress granule formation, structure, and function (Kedersha *et al.* 2000; reviewed in Anderson and Kedersha 2008). The three RNA recognition motifs (RRM) of TIA-1/TIAR proteins enable them to selectively bind to RNA, and their prion-like domain allows them to reversibly aggregate to form stress granules (Gilks *et al.* 2004; Kumar *et al.* 2008). Overexpression of the TIA-1 prion domain is sufficient to induce the formation of stress granules (Kedersha *et al.* 1999). Additional roles of TIA-1/TIAR proteins in regulating mRNA splicing and translation have been described (Förch *et al.* 2000; Dixon *et al.* 2003; Izquierdo *et al.* 2005; Waris *et al.* 2014). Embryonic and germ cell development are compromised in the absence or overexpression of *Tia-1* and *Tiar* in mice (Beck *et al.* 1998; Piecyk *et al.* 2000; Kharraz *et al.* 2010).

The germline of the nematode *Caenorhabditis elegans* provides a strong model system for analyzing translational regulation and other aspects of RNA biology (reviewed by Nousch and Eckmann 2013; Billi *et al.* 2014). *C. elegans* has three TIA-1/TIAR homologs: *tiar-1*, *-2*, and *-3* (WormBase release WS204). Among the three homologs, *tiar-1* is uniquely required for the induction of germ cell apoptosis under several stressful conditions (Silva-García and Navarro 2013). *tiar-1* mutant animals exhibit reduced longevity and small brood sizes and are hypersensitive to oxidative and UV stress (Silva-García and Navarro 2013; Rousakis *et al.* 2014). Additionally, TIAR-1 and -2 localize to germline-specific P granules in normal conditions (Jud *et al.* 2008; Silva-García and Navarro 2013) and to stress-induced granules in somatic tissues (Sun *et al.* 2011; Rousakis *et al.* 2014). However, the potential roles of TIA-1/TIAR-related proteins in protecting germ cells from stress are incompletely understood.

In this work, we investigated the function of TIAR-1 in *C. elegans* germline development under normal conditions and conditions of stress. We found that animals lacking *tiar-1* displayed chromosome nondisjunction during oogenesis under normal developmental conditions, resulting in infertility and embryonic lethality. When transiently exposed to a heat shock, early embryos stored in the uteri of the wild type and *tiar-1* null mutant adult hermaphrodites both exhibited a significant reduction in viability. However, embryonic lethality after heat shock was higher in *tiar-1* mutant animals than in the wild type. Remarkably, wild-type animals were able to recover completely from heat shock, such that their germ cells could produce primarily viable embryos. Conversely, *tiar-1* mutant animals continued to produce inviable embryos at an increased frequency, suggesting that *tiar-1* protects germ cells from heat-induced damage. The protective function of TIAR-1 correlated with its relocalization to cytoplasmic granules in the gonad following several stresses, including heat shock, starvation, and the inhibition of protein synthesis. We classified these stress-induced TIAR-1-containing granules into two groups based on their localization and formation dynamics, the first localized to the gonad core and the second to oocytes. The stress-induced TIAR-1-containing granules in both the gonad core and oocytes contained the CGH-1 DEAD-box helicase, which functions to regulate RNP dynamics (Hubstenberger *et al.* 2013, 2015). Gonad core and oocyte stress-induced granules displayed the following properties: they 1) dissolved upon restoration of normal conditions; 2) aggregated in the presence of puromycin, an enhancer of translational termination, even in the absence of environmental stress; and 3) dissolved in the presence of the polysome stabilizer cycloheximide. Nonetheless, *tiar-1* was required for the formation of granules in the gonad core but not in the oocytes. Gonad core granules did not seem to be needed for the germ cells to produce viable embryos after heat shock.

## Materials and Methods

### Strains

*C. elegans* strains were maintained at 20° on NGM-Lite and fed with the *Escherichia coli* strain OP50-1 (Brenner 1974; Sun and Lambie 1997). The following mutations were used: LGII–*tiar-1(tm361)*, *tiar-1(tn1543)*, *tiar-1(tn1545[tiar-1*::*s*::*tev*::*gfp])*, *unc-4(e120)*, *tra-2(q276)*, and *tiar-2(tm2923)*; LGIII–*unc-119(ed3)*; LGV–*fog-2(q71)*; and LGX–*tiar-3(ok144)*. The *gpIs1[Phsp-16.2*::*gfp]* heat-shock reporter was also used. The following rearrangements were used: *mIn1[dpy-10(e128) mIs14]* II and *mnC1dpy-10(e128) unc-52(e444)* II. *tiar-2(tm2923)* and *tiar-3(ok144)* mutations were confirmed by PCR and DNA sequencing. RN077 *tiar-1(tm361) xmSi02*[*Ptiar-1*::*tiar-1*::*gfp*::*tiar-1 3utr*; *Cbr-unc-119(+)*] II was constructed using single-copy insertion methodology (Frøkjær-Jensen *et al.* 2008, 2012). Details of strain constructions are available upon request. The complete list of strains used in this study, and their genotypes, are reported in Supplemental Material, Table S1.

To link the genetic marker *unc-4(e120)* to the *tiar-1(tm361)* allele, we crossed *tiar-1(tm361) xmSi02 [Ptiar-1*::*tiar-1*::*gfp*::*tiar-1 3utr*; *Cbr-unc-119(+)] II* males to *unc-4(e120) vab-1(dx31)* hermaphrodites. NonUnc, nonVab, and GFP-positive progeny were selected. Among the F2 generation, Unc, nonVab, and GFP-negative recombinant animals were selected. Finally, the lines carrying the *tiar-1(tm361)* allele were identified by PCR.

### Generation of tiar-1 alleles

CRISPR-Cas9 genome editing (Dickinson *et al.* 2013; Friedland *et al.* 2013) was used to generate the *tiar-1(tn1543)* deletion allele and the *tiar-1(tn1545[tiar-1*::*s*::*tev*::*gfp])* allele, which results from the insertion of S::TEV::GFP at the TIAR-1 C-terminus. We targeted a specific site in the last exon of *tiar-1* with a single-guide RNA (sgRNA) to be cut by Cas9. The protospacer-associated motif (PAM) for this sgRNA is at position 5,713,045 in the genome. To generate the *tiar-1* sgRNA, annealed primers (see Table S2) were inserted into the *Peef-1A.1*::*Cas9* empty sgRNA vector (pDD162; Addgene #47549) as described (Dickinson *et al.* 2013). Gibson assembly (New England BioLabs) was used to generate the repair templates with the pBluescript KS– vector as a backbone. The repair template for *tiar-1* deletion consisted of a 5′-upstream *tiar-1* homology arm, the *C. briggsae unc-119(+)* gene flanked by *loxP* sites, and a 3′-downstream *tiar-1* homology arm. The repair template for the *s*::*tev*::*gfp* insert consisted of the *tiar-1* gene, the *s*::*tev*::*gfp* insert, the *tiar-1* 3′-untranslated region (UTR), the *C. briggsae unc-119(+)* gene flanked by *loxP* sites, and a 3′-downstream region as a homology arm. To alter the PAM site from TGGA to TGCA in the repair template, site-directed mutagenesis was performed with the Q5 site-directed mutagenesis kit (New England BioLabs). To generate the mutants, the repair templates (10 ng/μl), the source of Cas9 (pDD162) containing the *tiar-1* sgRNA construct (50 ng/μl), pMA122 (10 ng/μl) (Addgene plasmid #34873), pCFJ104 (5 ng/μl) (Addgene plasmid #19328), pGH8 (10 ng/μl) (Addgene plasmid #19359), and pCFJ90 (2.5 ng/μl) (Addgene plasmid #19327) were microinjected into 60–80 young adult hermaphrodites from the strain HT1593 *unc-119(ed3)*. The hermaphrodites were recovered from the injection pads and cultured individually at 25° until starved cultures were produced. The plates were heat shocked at 34° for 3 hr, and the surviving nonUnc animals were isolated. One line for each one of the constructs was obtained. To remove the selectable marker, a mix of pDD104 (*eef-1A.1*::*Cre*; Addgene plasmid #47551; 50 ng/μl) and pCFJ90 (2.5 ng/μl) was microinjected into young adult hermaphrodites from both lines. For the *tiar-1*::*s*::*tev*::*gfp* strain, Unc animals were selected from the progeny. The selectable *C. briggsae unc-119* marker was removed from the strain, leaving the *gfp* Tag in frame with *tiar-1* coding sequences, followed by its 3′-UTR and one *loxP* scar. The resulting allele was confirmed by PCR and sequencing. We were unable to excise the *C. briggsae unc-119(+)* marker for the *tiar-1* deletion allele, so it remained in the *tiar-1* locus. Sequencing data indicates that the selectable *C. briggsae unc-119(+)* marker is placed in the *tiar-1* genetic locus, and *tiar-1* coding sequences were not detected by PCR. The resulting strains were outcrossed with N2 three times.

The plasmids used were a gift from Arshad Desai, Bob Goldstein, and Erik Jorgensen (Cheeseman and Desai 2005; Frøkjær-Jensen *et al.* 2008, 2012; Sando *et al.* 2013).

### Stress

Animals were grown at 20° or 24° on NGM-lite plates with NGM-lite seeded with OP50-1 bacteria until they were 1-d-old adults. The population was separated into stressed and control groups. For starvation, the animals were transferred to M9 containing cholesterol (5 μg/ml) and kept at 20° for 4 hr. For the control, animals were transferred to M9 containing cholesterol and freshly grown OP50-1 (a 1/20 dilution of a culture with a 0.66 absorbance at 600 nm). For heat shock, the animals were transferred to seeded plates, which were then sealed with Parafilm, and put into a controlled temperature water bath at 31° for 3 hr. Control (no stress) group plates were kept in the incubator at 20°. For stress recovery, the animals were returned to NGM-lite plates seeded with OP50-1 at 20°.

For drug treatments, freshly prepared stock solutions were added to S medium (Hope 1999) to a final concentration of 30 mM cycloheximide (Sigma-Aldrich, St. Louis, MO), or 15 mM puromycin (Sigma-Aldrich, St. Louis, MO). For experiments in which animals were fed in S medium, a 1/20 dilution of a bacterial culture having an absorbance at 600 nm of 0.66 was added. For starvation experiments, young adults were placed in S medium with (control), or without, OP50-1 and kept at 20° for 4 hr. For heat-shock experiments, young adults were cultured in S medium with OP50-1 and placed in the controlled temperature water bath at 31° for 3 hr, or kept at 20° (control).

### Quantitation of embryonic lethality after stress

Hermaphrodites were grown at 20 or 24°. They were cloned individually to plates at the mid-L4 stage; 18–20 hr later the young adult hermaphrodites were heat shocked at 31° for 3 hr in a controlled temperature water bath, or starved in M9 containing cholesterol (5 μg/ml) for 4 hr. Control groups were kept at the initial growth conditions. Immediately after the stress, animals were mounted without any anesthetic onto 2% agarose pads with M9 and observed under the microscope. The embryos in the uterus and the fully grown oocytes (–1 to –3) in each gonad arm of every hermaphrodite were counted. Then, animals were recovered on NGM-lite seeded plates at 20° and allowed to lay as many embryos as counted earlier, constituting group I. Since *tiar-1(0)* mutant hermaphrodites laid eggs more slowly than the wild type, *tiar-1(0)* mutant hermaphrodites were left on plates longer (usually 1–2 hr), until they laid a number of embryos corresponding to those counted prior to heat shock, plus those generated from fertilization of fully grown oocytes immediately thereafter. This allowed the group I embryos of both strains to be directly comparable. Afterward, the hermaphrodites from both strains were transferred to new plates and allowed to lay embryos for 12 hr, constituting group II. Once again, the hermaphrodites were transferred to new plates and allowed to lay embryos for another 24 hr, constituting group III. The embryonic lethality was determined as the percentage of embryos that did not hatch within 24 hr of being laid. In parallel, the embryonic lethality of hermaphrodites that were not heat shocked was scored as a nonstress control. Up to 30% of *tiar-1(tn1543)* and *tiar-1(tm361) unc-4(e120)* animals became strongly egg-laying defective (Egl) after heat shock, containing live hatchlings and dead embryos in their uterus. These Egl animals were censored from the study because it was not possible to accurately count embryos in the uterus once animals hatched internally and began consuming the parent.

### Immunostaining

To visualize germline granules, immunostaining against CGH-1 was performed as previously reported (Navarro *et al.* 2001). Briefly the gonads of 1-d-old animals were dissected, freeze-cracked, fixed in cold methanol for 1 min, and then in 3.3% paraformaldehyde for 30 min. For coimmunostaining with anti-CGH-1 and anti-GFP, the samples were fixed for only 18 min. Then, the samples were blocked with 30% normal goat serum (NGS; Sigma-Aldrich, St. Louis, MO) in PBT for 30 min. Primary antibody incubation was performed overnight at 4° with rat anti-CGH-1 (1:25; Navarro *et al.* 2001). Coimmunostaining used rabbit anti-CGH-1 (1:1000; Boag *et al.* 2005) and mouse anti-GFP (1:5000; A11120 from Molecular Probes, Eugene, OR). Secondary antibody incubations were performed for 1.5 hr at room temperature with Cy3-conjugated donkey anti-rat IgG (1:100; H+L; 112165003, Jackson ImmunoResearch, West Grove, PA), or with Alexa Fluor 594-conjugated anti-rabbit IgG and Alexa Fluor 488-conjugated anti-mouse IgG (1:100; H+L; A11001, Molecular Probes, Eugene, OR). To detect DNA, 1 ng/μl 4′9,6-diamidino-2-phenylindole (DAPI) was used. Vectashield (Vector laboratories, Burlingame, CA) was added to avoid photo bleaching before sealing the sample.

### Image acquisition and processing

The images of living animals, anesthetized with 0.01% tetramisole in M9, were acquired on a Nikon Eclipse E600 microscope equipped with an AxioCam MRc camera and Zeiss AxioVision software. The images of gonad granules were acquired on an Olympus FV10i confocal microscope with a 60x objective lens, NA = 1.2, a 1024 × 1024 size, 8x quality, and 2.0x confocal aperture. The images used to compare levels of GFP expression of the *Phsp-16.2*::*gfp* reporter transgene were taken with identical exposures.

Quantification of GFP fluorescence used Image J (1.50b, Wayne Rasband, National Institutes of Health). The whole area of each animal was selected, and then its integrated density was measured. The corrected total fluorescence was calculated as follows: Corrected Total Fluorescence (CTF) **=** integrated density – (selected area × mean fluorescence of three background readings).

### Statistical analysis

Brood size, embryonic lethality, and GFP fluorescence data were tested for equality of group variance. One-way ANOVA tests were performed for those datasets that had the same group variance. For datasets with unequal group variance, nonparametric multiple comparisons were performed using Dunn’s method, with the wild-type value as a control (JMP v9, Statistical Discovery, SAS). The data obtained from the “embryonic lethality after stress” assay was fitted to a Least Squares model (JMP v9, Statistical Discovery, SAS). The response evaluated was “embryonic lethality.” Each one of the tested individuals was identified, and the “individual” variable was added to the model as a random effect. The model predicted that the variables “group,” “stress condition,” and “genotype” and the interaction of the three had a significant effect on embryonic lethality. Tukey HSD was calculated to evaluate Least Square Means differences. For all tests *P* < 0.01 was considered as statistically significant.

### Data availability

Strains and sequences of DNA constructs will be provided upon request. The DNA sequences of the primers used to produce new *tiar-1* alleles with CRISPR-Cas9 genome editing are reported in Table S2.

## Results

### TIAR-1 promotes fertility and embryonic development

**Figure 1:**
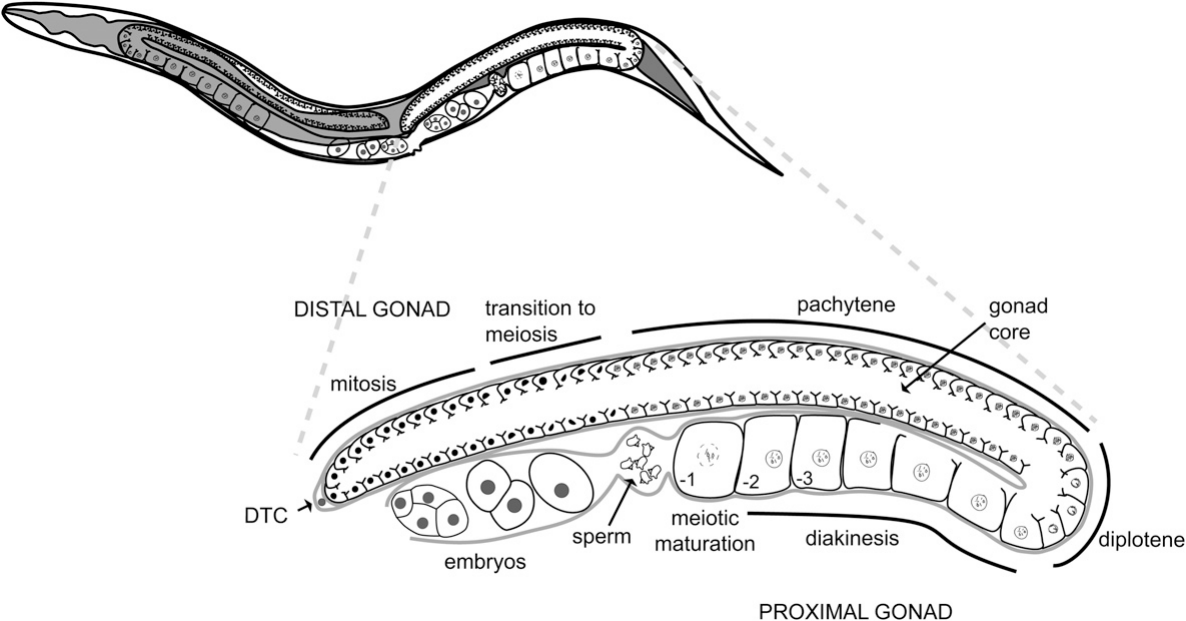
*C. elegans* adult hermaphrodite gonad. This model represents in detail the morphology of one of the two arms that compose the *C. elegans* adult hermaphrodite gonad. The upper picture represents a complete animal where the gonad and intestine (dark gray) are depicted. The bottom picture depicts one arm of the gonad zoomed in. Distal tip cell (DTC).

The *C. elegans* hermaphrodite gonad is composed of two identical U-shaped arms (Figure 1). Each gonad arm is a tubular structure in which most germ cells share a common core cytoplasm, forming a syncytium. The germline stem cells are located at the distal end of these gonad arms. As germ cells proliferate, they move proximally (closer to the uterus) and enter meiosis. During the L4 larval stage of development, the first ∼40 germ cells that enter meiosis differentiate into spermatocytes that produce ∼160 sperm, which enter a sperm storage compartment (spermatheca) at ovulation and are stored for self-fertilization. During adulthood, germ cells that enter meiosis differentiate into oocytes. Oocytes undergo meiotic maturation (entry into M phase of meiosis I from prophase), ovulation, and fertilization in an assembly-line-like fashion (Kim *et al.* 2013; Pazdernik and Schedl 2013).

**Figure 2:**
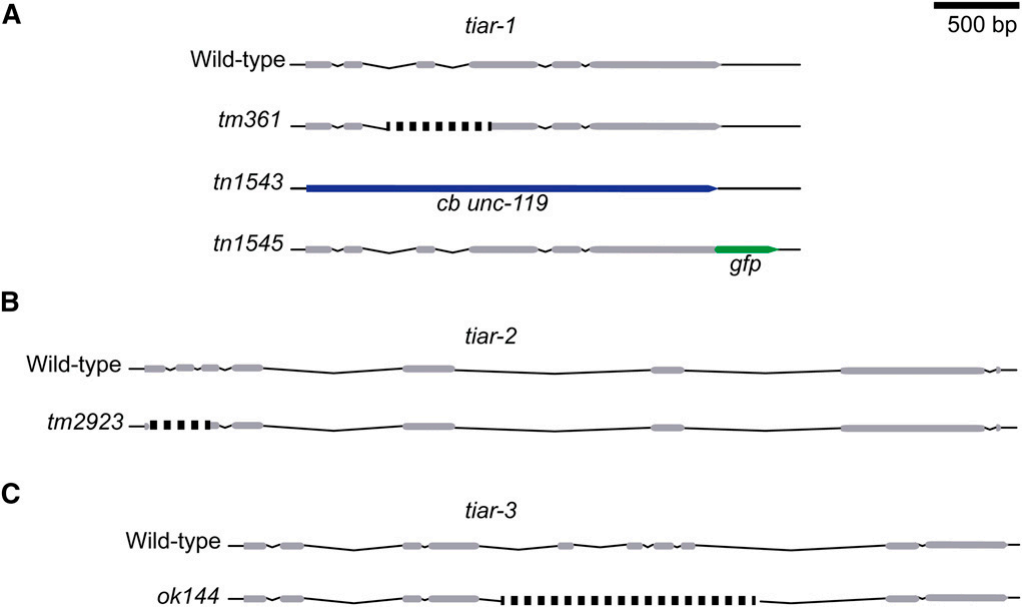
Alleles used to study the function of *tiar* genes in *C. elegans*. (A) The *tiar-1* wild-type allele comprises six exons (gray) and five introns (thin peaks). The *tiar-1(tm361)* allele is an out-of-frame deletion of 581 bp (dotted line). We generated a deletion and a GFP fusion allele using CRISPR-Cas9 genome editing. The strain *tiar-1(tn1543)* results from a deletion of the *tiar-1* open reading frame. In this strain, *tiar-1* sequences were replaced with the *C. briggsae unc-119* gene as a positive selection marker (blue). The strain *tiar-1(tn1545)* has an in-frame insertion of the tag S::TEV::GFP at the TIAR-*1* C- terminus (green). (B) The *tiar-2* wild-type allele comprises eight exons and eight introns. The *tiar-2(tm2923)* is a deletion of 336 bp (dotted line). (C) The *tiar-3* wild-type allele comprises 10 exons and nine introns. The *tiar-3(ok144)* is a deletion of 1531 bp (dotted line).

Previously, we reported that *tiar-1(tm361)* (Figure 2A) mutant hermaphrodites are temperature-sensitive sterile (Silva-García and Navarro 2013). However, in this study we found that *tiar-1(tm361)* adult hermaphrodites exhibit reduced fertility at 25° (76% infertility; Table S3) and that there is a linked mutation in the originally studied *tiar-1(tm361)* strain that exacerbates its infertility. We used genetic recombination (see *Materials and Methods*) to remove the enhancer mutation and to link *tiar-1(tm361)* to the commonly used genetic marker *unc-4(e120)*, which confers a backward locomotion defect and has no reported effect on fertility (Brenner 1974). In contrast to the originally studied *tiar-1(tm361)* mutant strain, which exhibits 76% infertility, *tiar-1(tm361) unc-4(e120)* mutant hermaphrodites exhibit 21% infertility (Table S3). Thus, we used the backcrossed *tiar-1(tm361) unc-4(e120)* strain for our studies hereafter. As described below, we independently isolated a *tiar-1* mutant allele using CRISPR-Cas9 genome editing (Figure 2A). This allele, *tiar-1(tn1543)*, exhibited 21% infertility at 25°, similar to the *tiar-1(tm361) unc-4(e120)* strain (Table S3).

We generated a new deletion allele [*tiar-1(tn1543)*; Figure 2A] by CRISPR-Cas9 genome editing (as in Dickinson *et al.* 2013). This allele results from a replacement of the entire *tiar-1* open reading frame with the *unc-119(+)* gene from *C. briggsae* as a selectable marker (Maduro and Pilgrim 1996). For simplicity, we refer to both *tiar-1(tm361) unc-4(e120)* and *tiar-1(tn1543)* alleles as *tiar-1(0)*. When grown at 20°, wild-type and *tiar-1(0)* hermaphrodites are fertile (Table 1). However, *tiar-1(0)* mutants produced smaller broods than the wild type at 20° (∼40–60% of wild type; Table 1). *tiar-1(0)* mutant animals exhibit appreciable embryonic lethality at 20° (∼10%; Table 1). Previous reports indicate that *tiar-1(tm361)* animals exhibit temperature sensitivity (Silva-García and Navarro 2013; Rousakis *et al.* 2014). Consistent with these results, *tiar-1(0)* mutant hermaphrodites grown constantly at 25° in our study exhibited low penetrance sterility, slower development, and additional somatic defects, such as protruding vulvae (Table S3; G. Huelgas-Morales and R. Navarro, unpublished results).

**Table 1:** *tiar-1(0)* mutants exhibit embryonic lethality and high-incidence of male progeny

Genotype	T	Brood Size	Embryonic Lethality (%)*^a^*	Males (%)*^b^*	*N*
Wild type	20°	281.9 ± 29.8	0.4 ± 0.5	0.06 ± 0.14	39
	25°	226.0 ± 35.9	0.5 ± 0.5	0.05 ± 0.17	19
	20–25° Shift	211.8 ± 35.9	0.6 ± 0.7	0.06 ± 0.19	39
*tiar-1(tn1543)*	20°	108.5 ± 26.4***	10.2 ± 10.0**	0.43 ± 0.80	12
	25°	22.9 ± 13.8***	22.7 ± 21.8***	2.01 ± 3.94	25
	20–25° Shift	33.9 ± 15.9***	20.5 ± 19.5***	1.87 ± 3.15	67
*tiar-1(tm361) unc-4(e120)*	20°	165.2 ± 40.3**	7.9 ± 5.6***	0.13 ± 0.41	21
	20–25° Shift	36.2 ± 43.7***	7.5 ± 12.3***	2.07 ± 6.36	50
*tiar-1(tn1543)/tiar-1(tm361) unc-4(e120)*	20°	129.2 ± 25.1***	4.8 ± 3.5***	0.14 ± 0.33	25
	20–25° Shift	24.9 ± 12.6***	31.6 ± 17.3***	1.13 ± 3.17	20
*tiar-2(tm2923)*	20°	271.8 ± 66.7	1.3 ± 2.3	0.08 ± 0.15	20
	20–25° Shift	181.1 ± 30.7	1.9 ± 2.2	0.34 ± 0.47	20
*tiar-3(ok144)*	20°	192.9 ± 28.3	1.1 ± 1.2	0.07 ± 0.2	15
	20–25° Shift	171.1 ± 32.8	5.5 ± 3.4	0.37 ± 0.37	15
*tiar-1(tn1543)*; *tiar-3(ok144)*	20°	105.2 ± 37.3***	9.2 ± 10.5***	0.55 ± 0.79	50
	20–25° Shift	32.4 ± 23.2*	44.0 ± 22.4***	2.44 ± 5.41	47
*tiar-1(tn1543) tiar-2(tm2923)*	20°	70.2 ± 31.5***	25.9 ± 15.9***	0.94 ± 1.26	27
	20–25° Shift	17.4 ± 17.2***	83.0 ± 30.3***	ND*^c^*	21
*tiar-1(tn1543) tiar-2 (tm2923)*; *tiar-3(ok144)*	20°	37.9 ± 18.6***	10.1 ± 10.9***	0.30 ± 1.61	30
	20–25° Shift	0.8 ± 2.4***	75.00 ± 50.0**	ND*^c^*	24
*fog-2(q71)* females *x tra-2(q276)* XX males	20–25° Shift	ND	ND	0.21 ± 0.51	18
*tiar-1(tn1543)*; *fog-2(q71)* females *x tra-2(q276)* XX males	20–25° Shift	ND	ND	1.88 ± 3.05	31
*tiar-1(tn1545[tiar-1*::*gfp])*	20°	252.8 ± 46.7	1.4 ± 2.2	0.08 ± 0.22	30
	20–25° Shift	136.6 ± 28.4	5.7 ± 6.3	0.17 ± 0.42	22

Hermaphrodites were incubated at a temperature (T) of 20 or 25° throughout their development. Then they were individually selected at the mid-L4 stage and transferred to new plates every 24 hr over the course of 3 d. Plates were scored for dead embryos, surviving progeny, and males. Embryos not hatching within 24 hr after being laid were considered dead. For upshift experiments, animals grown at 20° were shifted to 25° at the mid-L4 stage. Dunn’s test (wild-type values as control). *** *P* < 0.0001, ** *P* < 0.001.

a Percent of embryos among the progeny that did not hatch within 24 hr of being laid.

b Percent of male individuals among living progeny. The incidence of males was measured by scoring between 467 and 10,950 total animals.

c The progeny laid at 25° did not reach adulthood after 4 d.

To more directly assess fertility defects during adulthood, *tiar-1(0)* mutants were shifted from 20° to 25° during the L4 stage (mid-L4). In these upshifted *tiar-1(0)* animals, the defects increased in severity compared to animals grown at 20°. *tiar-1(0)* upshifted animals produced broods that were ∼17% the size of those of the wild type, and a fraction of their embryos did not survive (Table 1). *tiar-1(tn1543)* mutant hermaphrodites continually grown at 25° behave similarly to animals shifted to 25° at the mid-L4 stage (Table 1). This result suggests that the infertility is likely to arise from temperature-sensitive defects during oogenesis, which commences during the L4 stage, and continues into adulthood. Additionally, we analyzed *tiar-1(tn1543)/tiar-1(tm361) unc-4(e120)* heterozygous hermaphrodites and also observed a small brood size and embryonic lethality similar to those of *tiar-1(tn1543)* (Table 1). These results suggest that *tiar-1* promotes fertility and embryonic development.

Since *C. elegans* has three TIA-1/TIAR homologs, we also investigated the effects of *tiar-2* and *tiar-3* on fertility and embryonic development. To this end, we used *tiar-2(tm2923)* and *tiar-3(ok144)* mutants (Figure 2, B and C). The brood size of *tiar-2* and *tiar-3* mutant animals was slightly smaller than that of the wild type at both tested temperatures (Table 1). Both *tiar-2* and *tiar-3* mutant animals displayed a very slight increase in embryonic lethality compared to the wild type (Table 1); however, these differences were not statistically significant.

To test if *tiar-2* and *tiar-3* might have redundant roles with *tiar-1*, we studied double mutants. *tiar-1(tn1543)*; *tiar-3(ok144)* double mutants had a brood size similar to *tiar-1(tn1543)* mutants at the tested temperatures, but a higher proportion of the double mutant embryos did not survive when upshifted to 25° (Table 1). In contrast, *tiar-1(tn1543) tiar-2(tm2923)* double mutants had a smaller brood size than *tiar-1(tn1543)* mutants, and a much higher proportion of their embryos did not survive (83% when upshifted; Table 1). Thus, both *tiar-2* and *tiar-3* promote fertility and embryonic development, although *tiar-1* appears to play a more substantial role under the conditions tested than its paralogs.

Additionally, we studied *tiar-1(tn1543) tiar-2(tm2923)*; *tiar-3(ok144)* triple mutants and found that they produced very few progeny at both tested temperatures (Table 1). Embryonic lethality increased significantly upon upshift to 25° (∼75% embryonic lethality). However, the proportion of infertile triple mutant and *tiar-1(0)* single mutant animals was not significantly different (Table S3). Interestingly, *tiar-1(tn1543) tiar-2(tm2923)* and *tiar-1(tn1543) tiar-2(tm2923)*; *tiar-3(ok144)* double and triple mutant animals displayed a highly penetrant larval arrest when grown at 25°, suggesting that the three *C. elegans* TIAR paralogs play a redundant role in development. However, *tiar-1* appears to play the most evident role in promoting fertility and embryonic development.

### tiar-1(0) mutants exhibit an increased frequency of X-chromosome nondisjunction during oogenesis

*C. elegans* has two naturally occurring sexes; hermaphrodites have two sex (X) chromosomes and males have only one. Hermaphrodites produce ∼99.9% hermaphrodite progeny, with rare male progeny appearing to arise through X-chromosome nondisjunction (Hodgkin *et al.* 1979). Mutations that cause a high incidence of males (Him) phenotype cause meiotic defects that increase the frequency of X-chromosome nondisjunction (Hodgkin *et al.* 1979; reviewed by Hillers *et al.* 2015). Since we frequently observed males among the progeny of *tiar-1(0)* mutant hermaphrodites, we quantified their incidence. Wild-type animals produced 0.06% male progeny when grown at 20°, and when upshifted to 25° at the mid-L4 stage, or continually grown at 25° (Table 1). *tiar-1(0)* mutant hermaphrodites produced more males at both temperatures; the incidence of male progeny ranged from 0.1 to 0.4% at 20°, and from 1.1 to 2.1% at 25° (Table 1). Thus, we found that the incidence of males in the progeny of *tiar-1(0)* mutant animals was ∼2–7 times higher than in the wild type at 20°, and ∼10–35 times higher upon upshifting to 25°. The incidence of males in the progeny of *tiar-2* and *tiar*-3 mutant hermaphrodites was slightly higher than that seen in the wild type, but noticeably smaller than that observed in *tiar-1(0)* hermaphrodites (Table 1).

The high proportion of males produced by *tiar-1(0)* mutant hermaphrodites could potentially result from defects in spermatogenesis or oogenesis. Therefore, we assessed the relative contribution of defects in oogenesis and spermatogenesis to the *tiar-1(0)* mutant Him phenotype by evaluating the frequency of X-chromosome nondisjunction events when *tiar-1(0)* mutant oocytes are fertilized by *tiar-1(+)* sperm. To this end, we used *tra-2(q276)* mutants, which are XX but develop into fully functional males (Okkema and Kimble 1991), and *fog-2(q71)* hermaphrodites, which do not produce sperm and are often referred to as females (Schedl and Kimble 1988). When crossed to *tra-2* XX males, *tiar-1(tn1543)*; *fog-2(q71)* females produced ∼9 times more X0 males than *fog-2(q71)* females [Table 1; *tra-2(q276)* is recessive]. This result supports the hypothesis that X-chromosome nondisjunction during oogenesis significantly contributes to the *tiar-1(0)* Him mutant phenotype; however, we cannot eliminate the possibility that defects in spermatogenesis might also contribute.

### TIAR-1 associates with stress-induced granules in the germline

**Figure 3:**
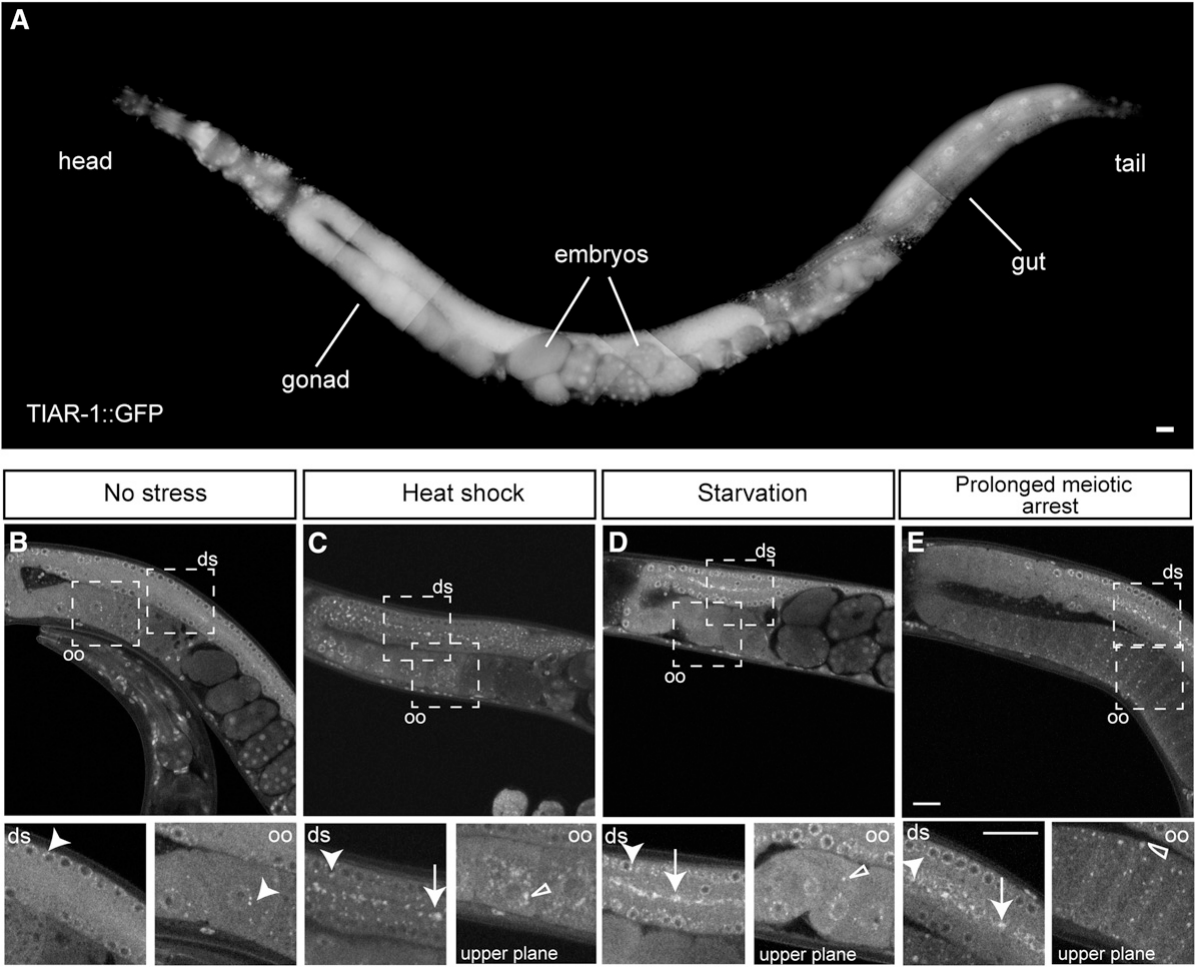
TIAR-1 associates with cytoplasmic granules during stress in the gonad. TIAR-1::GFP expression and localization were assessed in *tiar-1(tn1545)*. (A) 1-d-old *tiar-1*::*gfp* hermaphrodites were anesthetized and observed with the fluorescence microscope. The subcellular localization of TIAR-1::GFP in the gonad was observed under normal growth conditions (B), after exposure to heat shock (3 hr at 31°) (C), and after starvation (4 hr) (D). TIAR-1::GFP subcellular localization was also observed in *tiar-1(tn1545); fog-2(q71)* unmated females (E). These animals were imaged using confocal microscopy. Dotted squares indicate the zoomed-in areas. ds, distal gonad; oo, oocytes. Arrowheads indicate likely P granules, arrows gonad core granules, and empty triangles oocytes granules. Scale bars, 10 µm.

To study the expression of TIAR-1 *in vivo*, we used CRISPR-Cas9 genome editing to generate a C-terminal fusion of GFP to TIAR-1, creating *tiar-1(tn1545[tiar-1*::*gfp])* (Figure 2A). The brood size, embryonic lethality, and incidence of males in *tiar-1(tn1545[tiar-1*::*gfp])* strain displayed slight variations from those of the wild type; however, these differences were not statistically significant (Table 1). Importantly, the *tiar-1(tn1545[tiar-1*::*gfp])* strain was more fertile than the *tiar-1(0)* mutant strains at all temperatures examined (Table 1). These results indicate that the TIAR-1::GFP protein retains substantial function *in vivo*. Under normal growth conditions, we observed that TIAR-1::GFP is broadly expressed in both the soma and the germline, localizing to the cytoplasm and nuclei (Figure 3, A and B). In addition, TIAR-1::GFP localized to perinuclear foci that resemble P granules (arrowheads in Figure 3, A and B), consistent with previous findings (Silva-García and Navarro 2013).

In the *C. elegans* gonad, different kinds of granules are formed under a variety of stressful conditions (Boag *et al.* 2008; Jud *et al.* 2008; Noble *et al.* 2008; Paz-Gómez *et al.* 2014). Thus, we analyzed the localization of TIAR-1::GFP under several stressful conditions, including after exposure to heat shock (3 hr at 31°), starvation (no bacteria for 4 hr), and prolonged meiotic arrest in the absence of sperm [as in *tiar-1(tn1545)*; *fog-2(q71)* unmated females]. We found that TIAR-1::GFP associated with cytoplasmic granules under all of the tested stressful conditions (Figure 3, B–E). However, some TIAR-1::GFP remained diffuse in the cytoplasm, apparent P granules, and inside nuclei (Figure 3, B–E). TIAR-1::GFP associated with granules in two main regions: i) in the core of the gonad (Figure 3, C–E, distal gonad “ds,” arrows); and ii) in oocytes (Figure 3, C and E, oocytes “oo,” empty triangles). The TIAR-1::GFP-containing granules in the core of the gonad appear similar in shape and size in all the stressful conditions tested. However, TIAR-1-containing granules in oocytes display different characteristics depending on the nature of the stressful conditions. After heat shock, TIAR-1-containing oocyte granules are large and mainly perinuclear (Figure 3C, empty triangle). During prolonged meiotic arrest, TIAR-1-containing oocyte granules localized primarily to the cell cortex (Figure 3E, empty triangle). Following starvation, even when some TIAR-1-containing oocyte granules were visible, they were smaller and fewer in number (Figure 3D, empty triangle) than those observed under the other stressful conditions. The cytoplasmic granular localization of TIAR-1::GFP under stressful conditions is consistent with that observed for its mammalian homologs (Kedersha *et al.* 1999).

**Figure 4:**
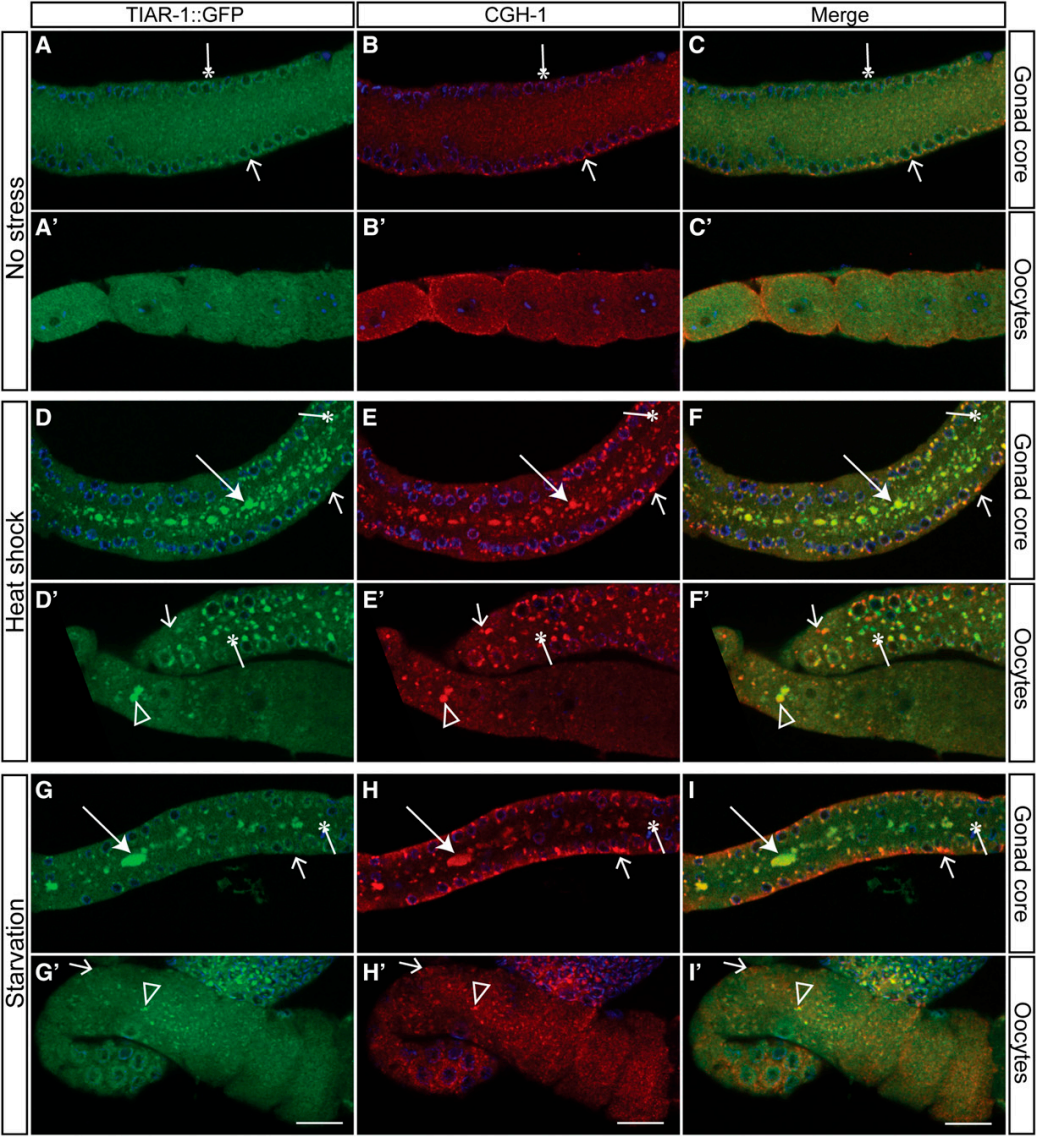
TIAR-1 and CGH-1 associate with large stress-induced granules in the gonad. *tiar-1(tn1545)* 1-d-old hermaphrodites were grown in normal conditions (no stress) (A–C’), exposed to heat shock (3 hr at 31°) (D–F’), or starved (4 hr without food) (G– I’). After the treatments, the animals were fixed and coimmunostained with anti-GFP, anti-CGH-1 (red), and DAPI (cyan) to visualize granules and DNA, respectively. The preparations were imaged using confocal microscopy. Note, TIAR-1 and CGH-1 associate with large stress-induced granules in the gonad core (panels A–I) and oocytes (panels A’–I’). However, some small granules appear to contain only TIAR-1 or CGH-1. Thin arrows indicate granules that appear to contain CGH-1 but not TIAR-1. Asterisks indicate granules that appear to contain TIAR-1 but not CGH-1. Arrows and empty triangles indicate granules in the gonad core and oocytes, respectively. Scale bar, 10 µm.

To explore whether the TIAR-1-containing granules in the gonad overlap with previously described stress-induced granules in *C. elegans*, we coimmunostained for TIAR-1::GFP and the CGH-1 DEAD-box RNA helicase, which associates with, and remodels, RNPs in the gonad under normal and stressful conditions (Navarro *et al.* 2001; Boag *et al.* 2008; Jud *et al.* 2008; Noble *et al.* 2008; Hubstenberger *et al.* 2013, 2015; Paz-Gómez *et al.* 2014). We observed that both proteins associate with large granules in the gonad core induced by heat shock or starvation (Figure 4, D–F and G–I, arrows). Likewise, the stress-induced granules in oocytes seem to largely represent overlapping structures (Figure 4, D’–F’ and G’–I’, empty triangles). However, in normal conditions, a few perinuclear granules appear to contain only TIAR-1 or CGH-1 (Figure 4, A–C, thin arrows and asterisks). These small granules, appearing to contain only one of the two proteins, became more evident under stressful conditions (Figure 4, D’–F’ and G’–I’, thin arrows and asterisks). Together, these results suggest that the TIAR-1-containing large stress-induced granules overlap with those previously described in *C. elegans* gonad, but a fraction of smaller cytoplasmic granules might have a different nature.

### Disruption of protein translation affects the formation of TIAR-1-containing stress-induced granules in the gonad

**Figure 5:**
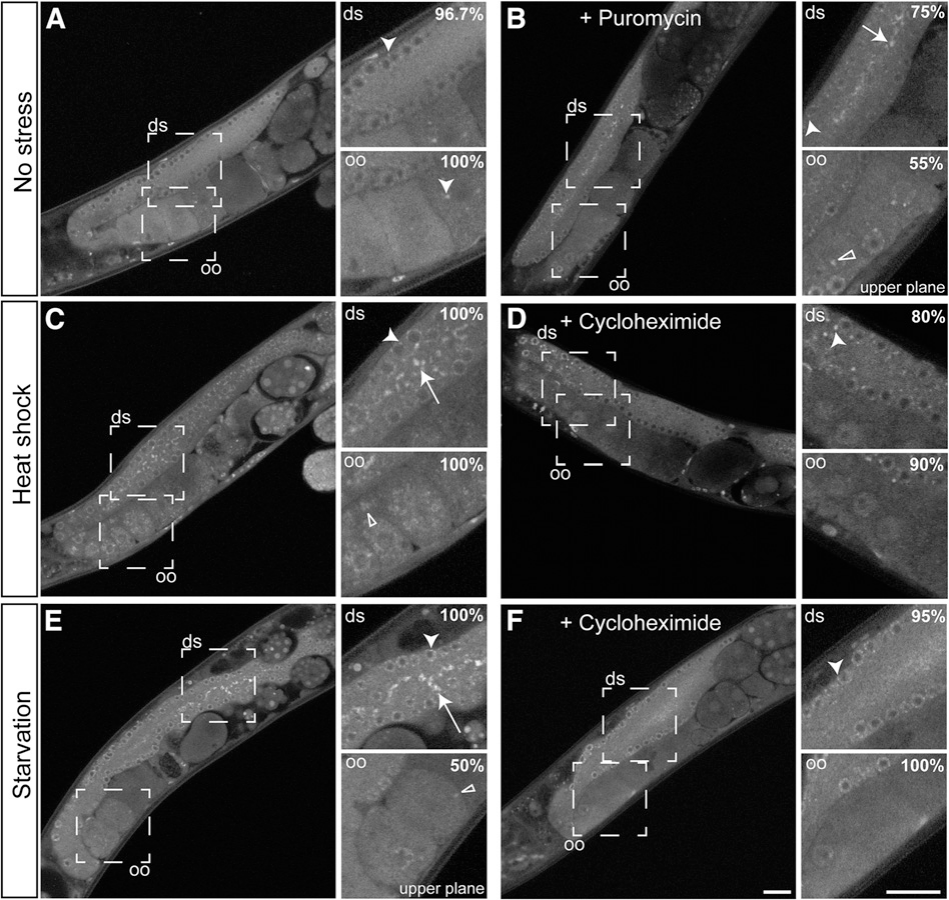
Cycloheximide treatment during stress impairs gonad granule formation. 1-d-old *tiar-1*::*gfp* hermaphrodites were grown at 20° in the absence of stress (A, B), or exposed to either heat shock (3 hr at 31°) (C) or starvation (4 hr) in liquid medium (see *Materials and Methods*) (E). *tiar-1*::*gfp* hermaphrodites were also incubated with 30 mM cycloheximide while being exposed to the heat shock (D) or starvation (F). Additionally, *tiar-1*::*gfp* hermaphrodites were treated with 15 mM puromycin in the absence of stress (B). After the incubation periods, the animals were mounted and imaged using confocal microscopy. Note, puromycin promotes granule formation in most of the nonstressed animals, while cycloheximide impairs granule formation in most of the stressed animals. Dotted squares indicate the zoomed-in areas; “upper plane” refers to a different confocal plane. ds, distal gonad; oo, oocytes. Arrowheads indicate likely P granules, arrows gonad core granules, and empty triangles oocytes granules. The percentage of animals with the depicted phenotype is shown. Scale bars, 10 µm.

Protein synthesis inhibitors that stabilize polysomes (*e.g.*, cycloheximide) hinder stress granule formation, and protein synthesis inhibitors that promote premature termination of translation (*e.g.*, puromycin) enhance stress granule assembly (Kedersha *et al.* 2000). We thus investigated whether protein synthesis inhibitors affect the formation of TIAR-1-containing granules. We treated *tiar-1(tn1545[tiar-1*::*gfp])* animals with either 30 mM cycloheximide or 15 mM puromycin under normal and stressful conditions. After heat shock, TIAR-1-containing granules in both the gonad core and oocytes formed in all of the nondrug-treated animals (Table 2 and Figure 5C, arrow and empty triangle). However, the fraction of heat-shocked animals that formed TIAR-1-containing granules in the gonad core and oocytes decreased substantially after cycloheximide treatment (Table 2 and Figure 5D). After starvation, TIAR-1-containing granules formed in the gonad core and in oocytes (Table 2 and Figure 5E, arrow and empty triangle). However, fewer of the starved animals formed TIAR-1-containing granules in both the gonad core and in oocytes following cycloheximide treatment (Table 2 and Figure 5F). After 3 hr of puromycin treatment, most animals formed gonad core granules, but fewer formed oocyte granules (Table 2). After 4 hr of puromycin treatment, the number of animals with TIAR-1-containing granules in the gonad core and oocytes increased (Table 2 and Figure 5B). This suggests that, during the puromycin treatment, granules form first in the gonad core and then in oocytes. Together, these results suggest that TIAR-1-containing granules in the *C. elegans* germline exhibit properties established for stress granules in mammalian systems.

**Table 2:** Cycloheximide treatment during stress impairs gonad granule formation

Conditions	Treatment	*tiar-1*::*gfp* Animals with Granules		
		Gonad core	Oocytes	*N*
Heat shock	None	100%	100%	40
	Cycloheximide	20%	10%	40
Starvation	None	100%	50%	22
	Cycloheximide	5%	0%	40
No stress	None	3.3%	0%	30
	Puromycin 3h	65.8%	10.5%	38
	Puromycin 4h	75%	55%	20

*tiar-1(tn1545)* 1-d-old hermaphrodites were exposed to the indicated conditions in liquid medium (see *Materials and Methods*). Then the animals were mounted and observed by confocal microscopy. The number of animals with visible TIAR-1::GFP granules in the gonad core and oocytes was quantified. The data shown is the average of at least two independent experiments.

**Figure 6:**
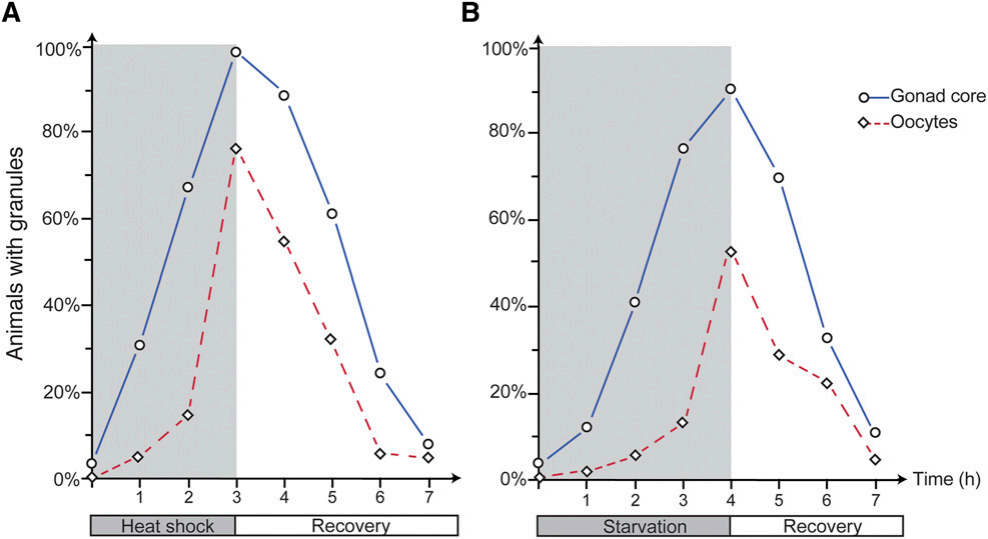
Gonad granules dissociate after stress recovery. *tiar-1*::*gfp* 1-d-old hermaphrodites were exposed to heat shock (3 hr at 31°) in agar plates (A) or starvation (4 hr with no bacteria) in liquid medium (B). Then they were recovered from stress (20° with bacteria). The animals were mounted, observed under the fluorescence microscope, and scored for presence of TIAR-1::GFP granules in the gonad core (blue solid line, circles) and in oocytes (red dotted line, diamonds) every hr during stress exposure and recovery. At least two independent experiments were conducted with *n* ≥ 50 for each condition and time point. The average percentage of animals with visible granules is depicted in the graphs.

Another feature of stress-induced granules is that they dissociate when normal conditions are restored (Kedersha *et al.* 2000). Thus, we analyzed TIAR-1-containing granules in the hermaphrodite gonad under stressful conditions and after recovery. During heat shock, the proportion of animals with granules in the gonad core and oocytes increased gradually, such that TIAR-1-containing granules were first observed 1 hr after heat shock (Figure 6A). After 3 hr at 31°, nearly all animals formed TIAR-1-containing granules in the gonad core, and most formed oocyte granules (Figure 6A). After recovery from heat shock, the proportion of animals with gonad granules decreased progressively. It took approximately 4 hr of recovery at 20° for the proportion of animals with granules to return to basal levels (Figure 6A). During starvation, the proportion of animals with TIAR-1-containing granules in the gonad core and oocytes increased less rapidly than during heat shock (compare Figure 6, A and B). It took approximately 4 hr of starvation for most animals to form TIAR-1-containing granules in the gonad core and in oocytes (Figure 6B). It took approximately 3 hr after refeeding for the proportion of animals with granules to return to basal levels (Figure 6B). In both stressful conditions, the TIAR-1-containing granules formed first in the gonad core and took longer to dissociate than those formed in the oocytes (Figure 6). Moreover, in both stressful conditions, the proportion of animals with TIAR-1-containing granules in the gonad core was always higher than those with granules in oocytes (Figure 6). These results suggest that TIAR-1-containing granules are dynamic structures that dissociate during recovery from stress, supporting the hypothesis that these structures behave like stress-induced granules in other organisms.

### tiar-1 is required for gonad core granule formation

**Figure 7:**
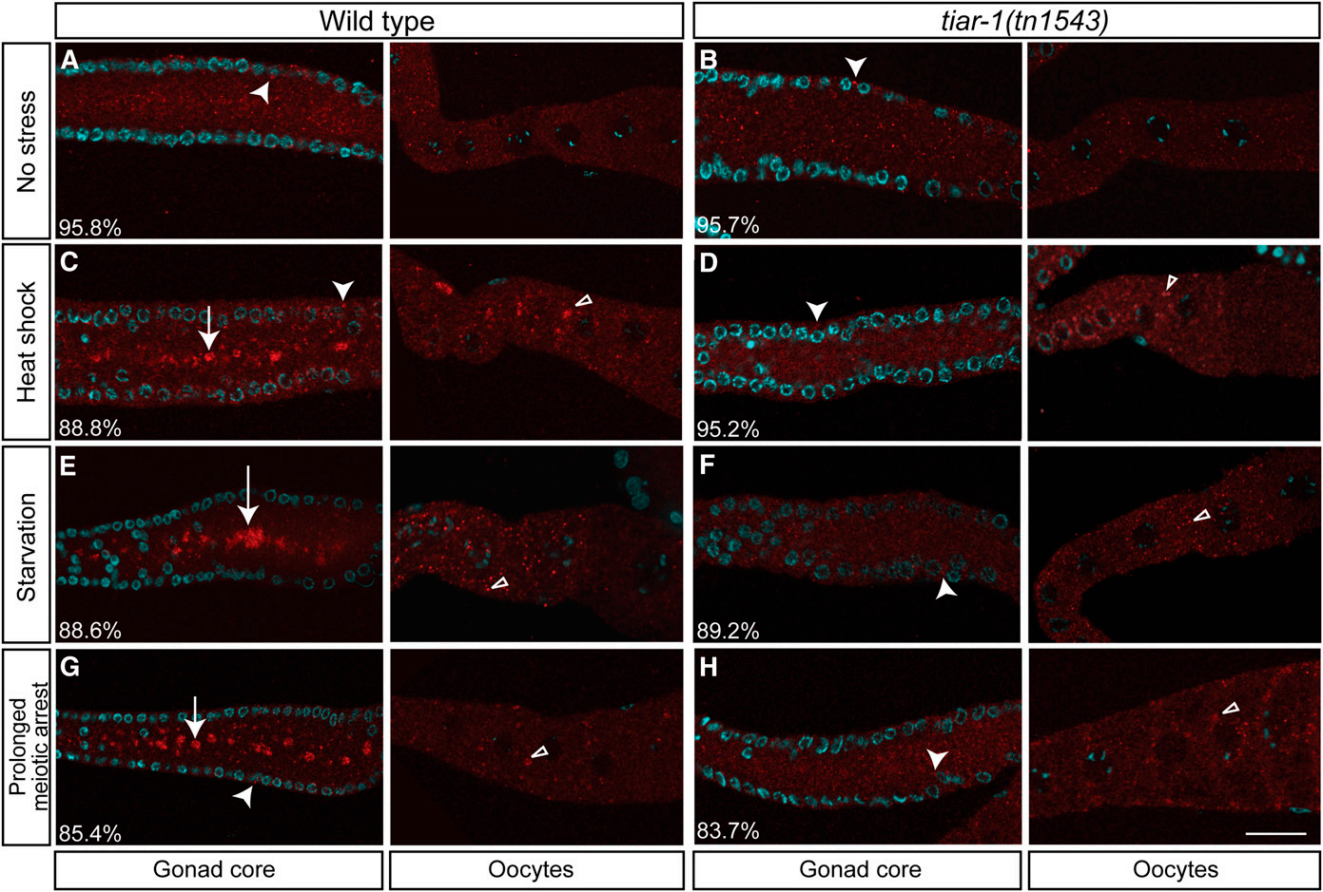
*tiar-1* is required for gonad core granule formation. Wild-type and *tiar-1(tn1543)* 1-d-old mutant hermaphrodites were grown in normal conditions (no stress) (A and B), exposed to heat shock (3 hr at 31°) (C and D), or starved (4 hr without food) (E and F). For prolonged meiotic arrest experiments, 1-d-old *fog-2(q71)* and *tiar-1(tn1543)*; *fog-2(q71)* unmated females were scored (G and H). After the treatments, the animals were fixed and immunostained with anti-CGH-1 (red) and DAPI (cyan) to visualize granules and DNA, respectively. The preparations were imaged using confocal microscopy. The average percentage of animals with the depicted phenotype was calculated from data obtained in at least three independent experiments. Note, granules are formed in the gonad core and oocytes of most wild-type animals. However, most of the *tiar-1* null mutants do not form granules in the gonad core, but do in the oocytes. Arrowheads indicate likely P granules, arrows gonad core granules, and empty triangles oocytes granules. Scale bar, 10 µm.

We evaluated whether *tiar-1* is required for formation of stress-induced granules in the gonad core and oocytes. Wild-type and *tiar-1(0)* animals were exposed to heat shock, starvation, and prolonged meiotic arrest conditions as described above. To visualize the stress-induced granules in the absence of TIAR-1, we immunostained for the CGH-1 DEAD-box RNA helicase, as described above (Figure 4). Under heat shock, starvation, and prolonged meiotic arrest conditions, wild-type animals formed large CGH-1-containing granules in both the gonad core and in oocytes (Table 3 and Figure 7, C, E, and G). In contrast, the majority of *tiar-1(0)* animals did not form gonad core granules under stressful conditions (Table 3 and Figure 7, D, F, and H). However, we observed that *tiar-2* and *tiar-3* mutant animals formed granules in the gonad core as often as wild-type animals (Table 3). Moreover, the proportion of *tiar-1(tn1543) tiar-2(tm2923)*, *tiar-1(tn1543)*; *tiar-3(ok144)* double, and *tiar-1(tn1543) tiar-2(tm2923)*; *tiar-3(ok144)* triple mutant animals that did not form gonad core granules was similar to that observed in *tiar-1* single mutant animals (Table 3). Interestingly, oocyte granules were still formed in *tiar-1(tn1543)* animals in all of the tested stressful conditions (Figure 7, D, F and H, empty triangles). These results demonstrate that *tiar-1* is required for formation of apparent stress granules in the gonad core, but that it might play a less critical role in the formation of granules in oocytes. Possibly, gonad core and oocyte granules form through different mechanisms. We cannot exclude a role for TIAR-1 in formation of oocyte granules under stressful conditions because TIAR-1 and CGH-1 do not exclusively colocalize under all conditions. Additionally, *tiar-2* and *tiar-3* did not seem to play a significant role in the formation of gonad core granules under the tested stressful conditions.

**Table 3:** *tiar-2* and *tiar-3* are not required for gonad core granule formation under stress

Genotype	Animals with Gonad Core Granules		
	No Stress	Heat Shock	Starvation
Wild type	4.2% (*n* = 119)	88.8% (*n* = 98)	88.6% (*n* = 79)
*tiar-1(tn1543)*	4.3% (*n* = 46)	4.8% (*n* = 42)	10.8% (*n* = 37)
*tiar-1(tm361) unc-4(e120)*	0% (*n* = 24)	15.2% (*n* = 33)	23.5% (*n* = 51)
*tiar-2(tm2923)*	0% (*n* = 37)	100% (*n* = 20)	100% (*n* = 24)
*tiar-3(ok144)*	7.1% (*n* = 42)	90.1% (*n* = 22)	100% (*n* = 27)
*tiar-1(tn1543) tiar-2(tm2923)*	0% (*n* = 28)	5.9% (*n* = 34)	6.5% (*n* = 31)
*tiar-1(tn1543)*; *tiar-3(ok144)*	0% (*n* = 45)	9.1% (*n* = 33)	23.5% (*n* = 34)
*tiar-1(tn1543) tiar-2(tm2923)*; *tiar-3(ok144)*	0% (*n* = 24)	15.2% (*n* = 33)	9.1% (*n* = 33)

1-d-old hermaphrodites of the indicated genotypes were kept under normal conditions or exposed to heat shock (3 hr at 31°) and starvation (4 hr without bacteria). Afterward, their gonads were dissected, fixed, and immunostained for CGH-1 (see *Materials and Methods*). The number of gonads with visible granules in the core was quantified and the average percentage is shown.

### tiar-1 protects female germ cells and embryos from heat shock

**Figure 8:**
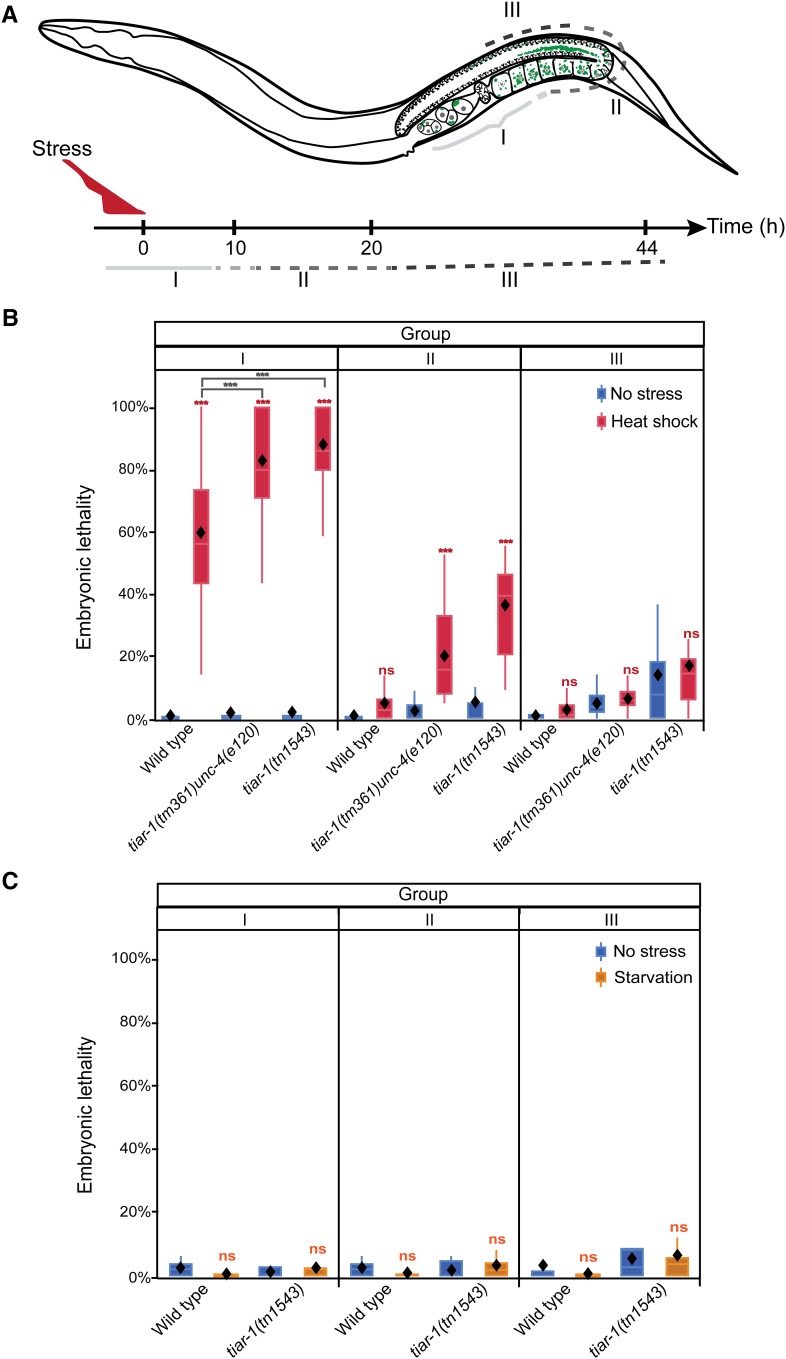
*tiar-1* protects embryos and germ cells from heat shock. Wild-type and *tiar-1(0)* young adult hermaphrodites were exposed to heat shock (31° for 3 hr), starved (4 hr), or kept at 20° on seeded plates as control (no stress). After stress, these animals were recovered to 20° on a seeded plate. Then their progeny were sorted into three groups: I) embryos that had been fertilized before or during stress, II) embryos that were produced shortly after stress, and III) embryos that were produced long after stress. (A) Scheme representing how the groups of embryos were assigned (see *Materials and Methods*). Embryonic lethality was quantified for all groups after heat shock (B) and starvation (C). Embryos not hatching within 24 hr of being laid were considered inviable. The graphs show the data obtained from three independent replicates. The boxes represent the 25–75% interquartile range, the diamonds the mean value, and the whiskers extend from the upper to the lower values. The *** (*P* < 0.001) and ns (nonsignificant) symbols placed on top of the boxes are comparisons between no-stress and stress groups, whereas the ones placed above them are comparisons between the stress groups. Least square means, Tukey HSD test.

Since TIAR-1-containing granules form in the germline under conditions of stress, we tested whether TIAR-1 might serve a protective function during gametogenesis or embryonic development. Thus, we exposed wild-type and *tiar-1(0)* mutant animals to heat shock (3 hr at 31°) and starvation (no bacteria for 4 hr) and analyzed the impact on embryonic viability at various times after alleviation of the stress (20° with bacteria). This analysis takes advantage of the spatially and temporally graded distribution of germline nuclei in the adult hermaphrodite gonad, with the proliferative stem cell population located distally, and developing oocytes progressing through the stages of meiotic prophase proximally (Figure 8A and Figure 1). At 20°, meiotic prophase during oogenesis takes approximately 54–60 hr (Jaramillo-Lambert *et al.* 2007). Because embryos are produced in an assembly-line-like fashion, we were able to distinguish between the effects of stress on full-grown oocytes and embryos from those on developing oocytes that were at earlier stages of meiotic prophase during the stress. This allowed for the sorting of the progeny of the stressed animals into three groups (see *Materials and Methods*). Group I comprised two classes of progeny: 1) embryos that were formed during the heat stress and that were already in the uterus; and 2) embryos formed shortly after the heat stress through the fertilization of full-grown diakinesis-stage oocytes (Figure 8A). Group II comprised embryos laid between 12 and 24 hr after stress, and group III comprised those laid between 24 and 48 hr after stress (Figure 8A). Group III likely corresponds to oocytes developing at earlier stages of meiotic prophase during the stress than those of group II. Whether group III also includes premeiotic germ cells subjected to stress is uncertain; however, a consideration of cell-cycle kinetics of *C. elegans* germline stem cells (Fox *et al.* 2011; Fox and Schedl 2015) and the 54–60 hr duration of meiotic prophase during oogenesis (Jaramillo-Lambert *et al.* 2007) suggests this might not be the case. Embryonic lethality was determined for each experimental group and the respective controls.

We observed that *tiar-1(0)* mutant adults display a slower egg-laying rate than the wild type; however, we accounted for this difference by collecting the embryos that were in the uterus, and those that were newly formed from fertilization of the –1 to –3 oocytes, such that group I progeny from both strains are directly comparable (see *Materials and Methods*). Because of this difference in egg-laying rate, comparison of groups II and III between *tiar-1(0)* mutants and the wild type must be viewed as an approximation. Thus, our analysis did not focus on this specific comparison. First, we analyzed the effects of heat shock on the wild type. We found that most of the wild-type embryos from group I did not survive the heat shock (Figure 8B). However, the majority of wild-type embryos from groups II and III survived (Figure 8B). This result indicates that wild-type developing oocytes at earlier stages of meiotic prophase are protected from the detrimental effects of heat shock. Either these germ cells are protected from the deleterious effects of heat shock, or they possess the means to repair the resulting cellular damage (or both). Similarly, the vast majority of *tiar-1(0)* mutant embryos from group I did not survive heat shock (both *tiar-1(tn1543)* and *tiar-1(tm361)* were examined; Figure 8B). However, analysis of the embryonic lethality observed in group I embryos suggests that *tiar-1(0)* mutants are more susceptible to heat stress than the wild type (Figure 8B). In this experiment, recovery from heat shock was at 20°, a temperature at which *tiar-1(0)* mutants exhibit ∼10% total embryonic lethality in the absence of heat shock (Table 1 and Figure 8B). Nonetheless, sorting embryos within broods of nonstressed animals revealed that *tiar-1(0)* mutant animals exhibit slightly higher embryonic lethality as they age (Figure 8B). Thus, the greater susceptibility of group I *tiar-1(0)* mutant embryos compared to the wild type suggests that *tiar-1* plays an important role in protecting diakinesis-stage oocytes and embryos from the deleterious effects of heat stress. Likewise, group II *tiar-1(0)* embryos exhibited appreciable embryonic lethality after heat shock (Figure 8B); however, the degree of embryonic lethality was less than that observed for group I. This result suggests that developing oocytes at earlier stages of meiotic prophase possess *tiar-1*-dependent and *tiar-1*-independent mechanisms to protect from heat stress. By contrast, *tiar-1(0)* group III embryos exhibited embryonic lethality no different than that of nonstressed animals (Figure 8B). This result suggests that *tiar-1*-independent mechanisms are sufficient to protect germ cells composing group III from the deleterious effects of heat stress. The resolution of this experiment is unable to pinpoint the precise stages of germ cell development at which *tiar-1*-independent mechanisms are sufficiently protective. Nonetheless, the recovery of viability in *tiar-1(0)* group III embryos indicates that the *tiar-1(0)* animals were not irreversibly damaged by heat shock. Since the sperm were formed in the L4 stage prior to heat shock, sperm appear less susceptible to heat shock than developing oocytes. In contrast to heat shock, starvation did not increase embryonic lethality in either the wild type or *tiar-1(0)* mutants (Figure 8C).

Although *tiar-1* is needed for the increase in germline apoptosis caused by starvation (Silva-García and Navarro 2013), *tiar-1*-independent mechanisms are sufficient to protect the germline from starvation. In response to starvation in the L4-larval stage, adult hermaphrodites can undergo adult reproductive diapause (ARD), during which germline stem cells are preserved for gametogenesis after refeeding (Angelo and Van Gilst 2009; Seidel and Kimble 2011). We found that *tiar-1* was dispensable for ARD (G. Huelgas-Morales, C. Silva-García, D. Greenstein, and R. Navarro, unpublished results). Taken together, these results suggest that 1) *tiar-1* is important to protect germ cells and embryos from heat stress, and 2) there are different mechanisms to protect germ cells and embryos from heat shock and starvation.

### tiar-1 protects female germ cells and embryos from heat shock independently of large gonad core granules

**Figure 9:**
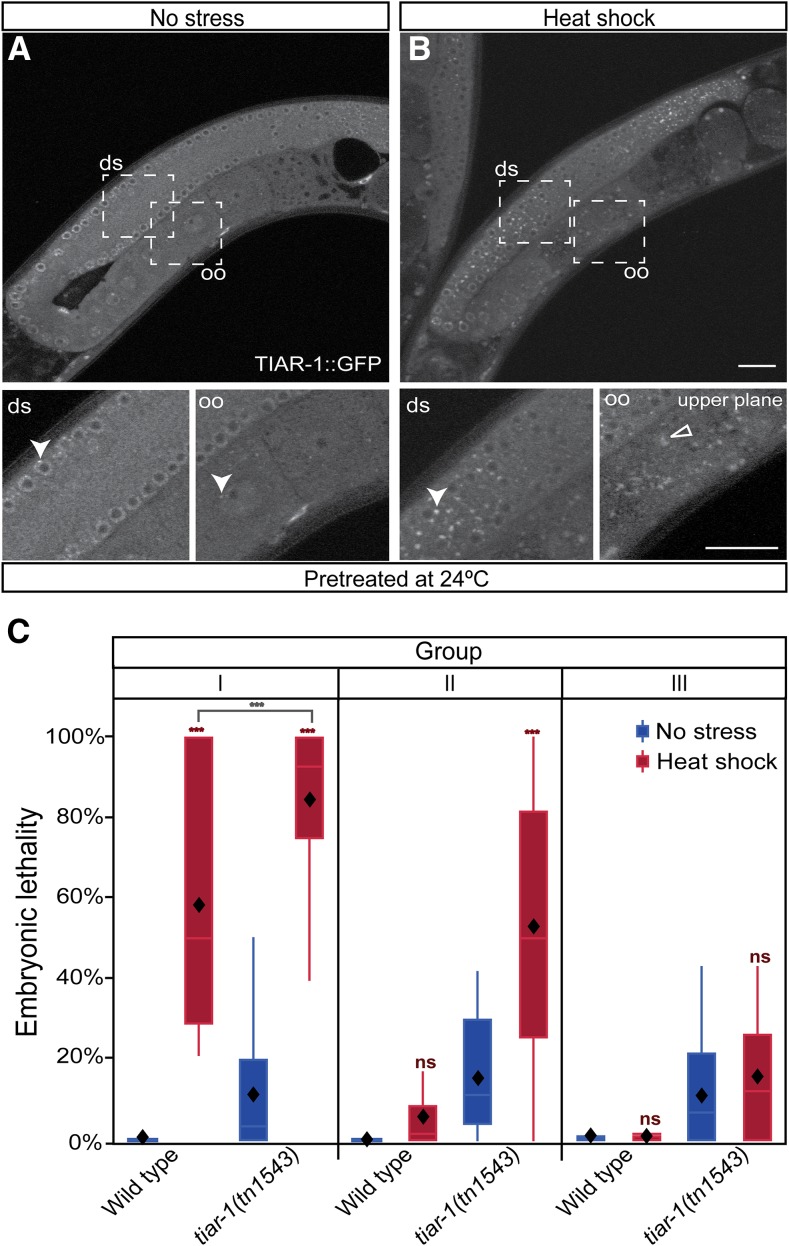
*tiar-1* protects female germ cells and embryos from heat shock independently of gonad core granules. Pretreated *tiar-1*::*gfp* animals (grown at 24°) were imaged using confocal microscopy. Representative images show gonads of animals (A) not exposed to stress, and (B) exposed to heat shock (31° for 3 hr). The photomicrograph in (B) is representative of animals that formed very small granules in the core gonad and normal granules in oocytes. However, most of the pretreated animals did not form core granules after the heat shock (Table 4). Arrowheads indicate likely P granules and empty triangles oocytes granules. Scale bar, 10 µm. (C) Pretreated wild-type and *tiar-1(tn1543)* hermaphrodites were exposed to heat shock (3 hr at 31°) or kept at 20° as a control. After, their progeny were sorted into three groups (as in Figure 7A). Embryonic lethality was quantified for all the groups (C). Embryos not hatching within 24 hr after being laid were considered inviable. The graphs show the data obtained from three independent replicates. The *tiar-1(tn1543)* animals included in this experiment were representative of the total population (see Table 4); we did not prescreen or exclude the small fraction of animals containing small granules in the gonad core. The boxes represent the 25–75% interquartile range, the diamonds the mean value, and the whiskers extend from the upper to the lower values. The *** (*P* < 0.001) and ns (nonsignificant) symbols placed on top of the boxes are comparisons between no-stress and stress groups, whereas the ones placed above them are comparisons between the stress groups. Least square means, Tukey HSD test.

The analysis above shows that heat stress induces the formation of TIAR-1-containing granules and that *tiar-1* protects developing oocytes from the detrimental effects of heat stress. Therefore, we investigated whether the protective function of TIAR-1 might involve granule formation. In the course of our experiments, we found a condition in which many heat-shocked animals did not form large gonad core granules. When wild-type animals are grown at 20° and then heat shocked (31° for 3 hr), 85–100% of them form large gonad core granules (Table 2 and Table 4, and see Figure 3C, arrow). However, when these animals were grown at 24° (from hatching until adulthood) and then heat shocked (31° for 3 hr), the formation of large granules in the gonad core was reduced. For simplicity, we refer to the animals grown at 24° as pretreated. We found that only 3% of the pretreated wild-type animals formed large CGH-1-containing granules, 22% of them formed small and scattered granules, and 75% did not form gonad core granules at all (two replicates, *n* = 32). We confirmed this finding on pretreated *tiar-1(tn1545[tiar-1*::*gfp])* animals (Table 4; Figure 9B depicts animals that formed the small granules). Note that the formation of oocyte granules remained unaffected in these conditions (Figure 9B).

**Table 4:** Large TIAR-1::GFP granules in the gonad core do not form in heat-shocked animals that were pretreated at 24°

	Control at 20° (*n* = 35)	Heat Shock, Animals Grown at 20° (*n* = 65)	Heat Shock, Animals Pretreated at 24° (*n* = 89)
Gonad core granules	0%	85%	7%
Small gonad core granules	6%	14%	39%
No gonad core granules	94%	1%	54%

*tiar-1(tn1545)* hermaphrodite animals were grown at 20 or 24°. When they were 1-d-old adults, they were exposed to the indicated conditions on agar medium. Then the animals were mounted and observed with the fluorescence microscope for gonad core granule formation. The number of animals with visible granules in the gonad core was quantified for at least two independent replicates and the average percentage is shown.

Pretreated wild-type animals were then exposed to heat shock, and their progeny were sorted into three groups and scored for embryonic lethality using the same sorting procedure described above. The results obtained for pretreated animals were similar to those for animals grown at 20°. We found that 58% of the pretreated wild-type embryos from group I did not survive heat shock (Figure 9C). However, the embryos from groups II and III did survive (Figure 9C). Thus, under conditions in which the majority of the pretreated heat-shocked animals did not form large gonad core granules (Figure 9B), most wild-type embryos from groups II and III survived (Figure 9C). This indicates that the large gonad core granules might be dispensable for germ cell protection under the tested circumstances. We cannot rule out the possibility that the small granules that are formed in some of the pretreated animals (Figure 9B) are sufficient to protect germ cells so they can later develop into viable embryos. Pretreated *tiar-1(tn1543)* animals displayed embryonic lethality similar to that of *tiar-1(0)* animals grown at 20° (84.5% for group I and 52.5% for group II). However, embryos from group III were no different to those not exposed to heat stress (Figure 9C). These results might indicate that TIAR-1 can protect germ cells and embryos from heat stress independently of its role in gonad core granule formation.

**Figure 10:**
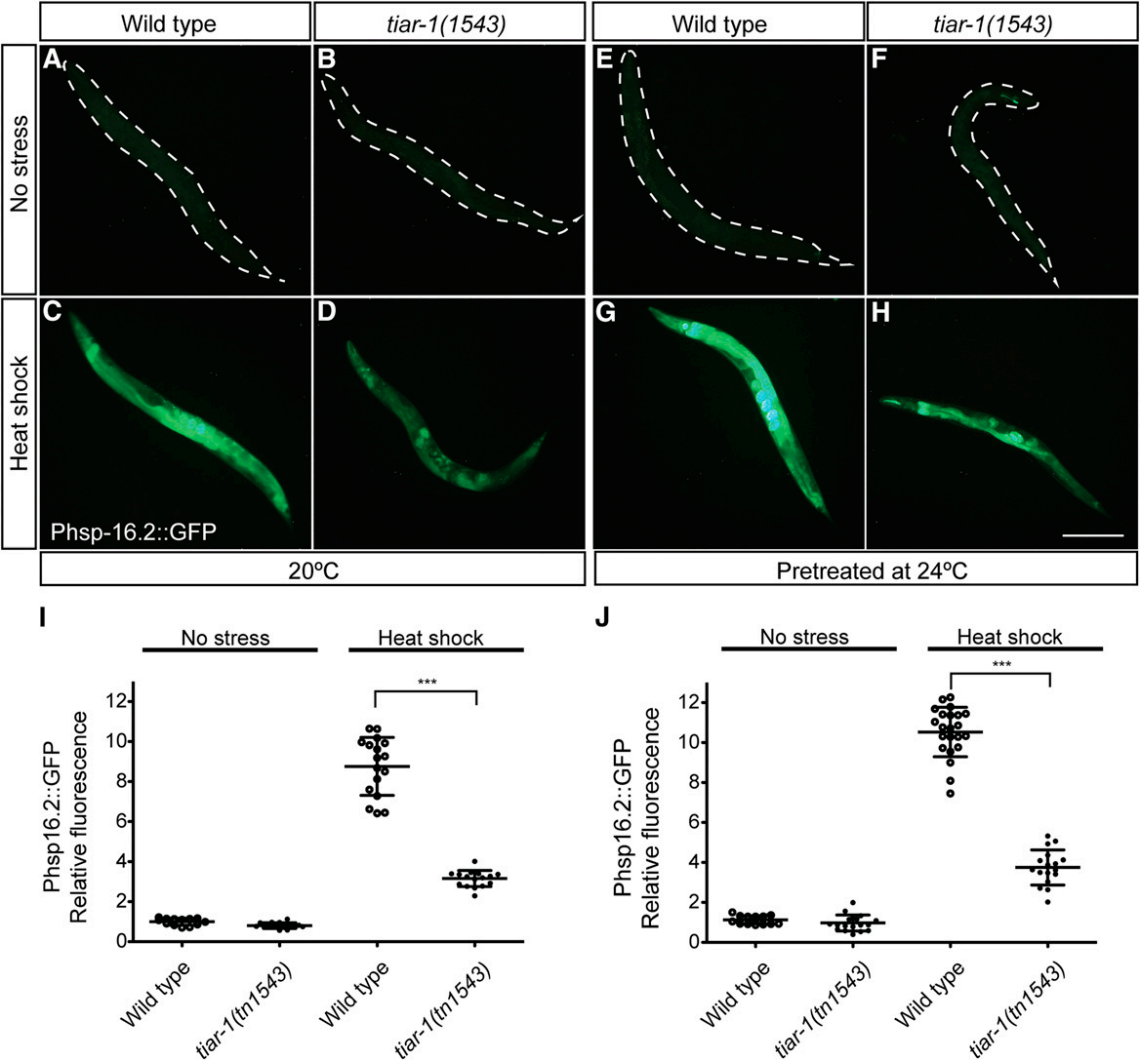
*tiar-1(tn1543)* mutant animals respond weakly to heat shock. Expression of *hsp-16.2*::*gfp* was assessed in *tiar-1(1543)* animals and normal controls. Strains carrying the *gpIs1[hsp-16.2*::*gfp]* insertion were grown either at 20° or pretreated at 24°. Then these animals were cultured under normal conditions (no stress) (A, B and E, F) or exposed to heat shock (3 hr at 31°) (C, D and G, H). Afterward, the animals were mounted and imaged using fluorescence microscopy. Photomicrographs of representative animals for each strain and condition were taken using the same exposure settings. The fluorescence of GFP (arbitrary units) was quantified for each of the genotypes and conditions. The values were normalized relative to the fluorescence of wild-type animals, grown at 20° in normal conditions. The data of one of three independent replicates with similar results are shown. One-way ANOVA *** *P* < 0.001.

Another possibility is that pretreated animals do not sense and/or respond to stress in the same way that animals grown at 20° do. To test this possibility, we evaluated the heat-shock response by assessing the expression of the transgene *gpIs1[Phsp-16.2*::*gfp]*, which has been used as a biomarker for thermotolerance (Rea *et al.* 2005). In normal conditions, wild-type animals showed little to no expression of *Phsp-16.2*::*gfp*, independently of the temperature at which they were grown (Figure 10, A and E). However, after heat shock, *Phsp-16.2*::*gfp* expression increased nine times in animals grown at 20° (Figure 10, C and I). When the animals were pretreated and then heat shocked, the transgene expression increased up to 10 times (Figure 10, G and J). These results indicate that pretreated animals still sense and respond to heat shock. To test if *tiar-1(0)* mutant animals sense and respond to heat shock, we evaluated *Phsp-16.2*::*gfp* expression in a *tiar-1(tn1543)* mutant background. In normal conditions, *tiar-1(tn1543)* showed little to no expression of *Phsp-16.2*::*gfp*, both when grown at 20° and when pretreated (Figure 10, B and F). Interestingly, when we exposed *tiar-1(tn1543)* to heat shock, the animals did not show the pronounced increase in *Phsp-16.2*::*gfp* that wild-type animals do. When *tiar-1(tn1543)* animals were grown at 20°, the expression increased only three times, compared to the nine fold increase observed in the wild-type control (Figure 10, D and I). When *tiar-1(tn1543)* mutant animals were pretreated at 24° and then heat shocked, *Phsp-16.2*::*gfp* expression increased only 3.5 times, compared to the 10-fold increase in the wild-type control (Figure 10, H and J). These results suggest that *tiar-1(tn1543)* mutants exhibit an impaired heat-shock response.

## Discussion

TIA-1 and TIAR are key proteins for mRNA regulation and stress granule formation in mammalian cells, as well as for fertility and embryonic development in mice (for review see Waris *et al.* 2014). Prior to this work, the potential involvement of TIA-1/TIAR proteins in protecting germ cells from stress was unclear. In this work, we show that *tiar-1* plays an important role in fertility and embryonic development, not only under normal conditions, but also during heat shock. Interestingly, the *tiar-1*-dependent large gonad core granules induced by heat stress appeared not to be required to protect germ cells and embryos from heat shock. Our study suggests that germ cells possess *tiar-1*-dependent and *tiar-1*-independent mechanisms to protect the germline from a variety of stresses, including heat shock and starvation.

### tiar-1 is important for embryonic viability

The TIA-1/TIAR family of proteins plays an important role in fertility. In mice, *Tiar* disruption results in incompletely penetrant background-dependent embryonic lethality (Beck *et al.* 1998; Piecyk *et al.* 2000). Surviving *Tiar* null mutant mice produce few primordial germ cells, but these germ cells do not populate the gonads, leading to male and female sterility (Beck *et al.* 1998). In contrast, *Tia-1* null mice are fully fertile (Piecyk *et al.* 2000). In *C. elegans*, it had been reported that a putative loss-of-function *tiar-1* allele, *tm361*, causes temperature-sensitive sterility (Silva-García and Navarro 2013; Rousakis *et al.* 2014). In this work, we found that *tiar-1* null mutants can be fertile at all temperatures at which *C. elegans* is typically cultivated, but that a closely linked mutation likely exacerbates the infertility phenotype in the original *tiar-1(tm361)* strain. Further, we verified a role for *tiar-1* in fertility by generating a new loss-of-function allele, *tiar-1(tn1543)*, using CRISPR-Cas9 genome editing. *tiar-1(0)* mutant animals were fertile at 20°, but ∼20% of them were infertile when grown at 25° (Table S3). Interestingly, the loss of the three *tiar* paralogs (*tiar-1–3*) did not lead to a substantial decrease in fertility. Importantly, the brood size of single *tiar-1*, double, and, particularly, triple mutant animals was significantly smaller than that of the wild type. Even when these mutant animals produce gametes, their fertility is severely compromised. Although *tiar-1*, the closest homolog to the mammalian genes, has the most evident role in *C. elegans* fertility, our results suggest that *tiar-2* and *tiar-3* are redundant with *tiar-1* to some degree.

The TIA-1/TIAR-family of proteins is also important for embryonic development. Both *Tiar* and *Tia-1* null mutant mice result in incompletely penetrant embryonic lethality (50–100%), as does *Tiar* overexpression (Beck *et al.* 1998; Piecyk *et al.* 2000; Kharraz *et al.* 2010). Moreover, mice lacking both *Tia-1* and *Tiar* die before embryonic day 7, which indicates that both of these proteins play important roles in embryonic development (Piecyk *et al.* 2000). In this work we obtained similar results to what has been reported for mice; the loss of *tiar-1* causes partially penetrant and temperature-sensitive embryonic lethality and the simultaneous loss of *tiar-1–3* further increases embryonic lethality in *C. elegans* (Table S3). Our observation of defects in meiosis during oogenesis provides a plausible explanation for this embryonic lethality. We observed a high incidence of males in *tiar-1(0)* mutants, which results from the nondisjunction of X chromosomes (Table 1). The precise role of TIAR-1 in oocyte meiosis and embryonic development remains to be determined.

### TIAR-1 forms stress-induced granules in the gonad

In response to stress and prolonged meiotic arrest, cytoplasmic granules are formed in the gonad core and oocytes of *C. elegans* adults (for review, see Schisa 2014). Both kinds of granules contain mRNA and RNA-binding proteins, such as CGH-1, PUF-5, VBH-1, CAR-1, OMA-1, and MEX-3, as well as Dicer (Boag *et al.* 2008; Jud *et al.* 2008; Noble *et al.* 2008; Beshore *et al.* 2011; Schisa 2012; Paz-Gómez *et al.* 2014; Spike *et al.* 2014). Poly(A) mRNA, as well as a few specific mRNAs, have been observed to localize to RNP granules in the gonad (Schisa *et al.* 2001; Noble *et al.* 2008; Spike *et al.* 2014). For example, *rme-2* mRNA was found to be associated to the granules only where it is translationally repressed (gonad core), but was not associated to the granules where it is translated (*i.e.*, in oocytes; Noble *et al.* 2008). Besides, at least one protein has been described to associate with oocyte but not gonad core RNP granules: DCAP-2 (Noble *et al.* 2008). In this work, we demonstrated that TIAR-1 associates with both groups of granules during heat shock, starvation, and prolonged meiotic arrest conditions, which enabled us to further study the behavior of these granules.

Mammalian stress-induced granules are dynamic structures that depend on translation (Kedersha *et al.* 2000). In *C. elegans*, TIAR-1 granules in somatic cells dissociate after recovery from stress as well (Rousakis *et al.* 2014). In this work, we provide further evidence showing that the stress-induced granules in the *C. elegans* gonad behave like their mammalian counterparts. The TIAR-1-containing stress-induced granules in both the gonad core and oocytes also dissociated after recovery from stress (3 hr after starvation and 4 hr after heat shock). Similarly, oocyte granules induced by prolonged meiotic arrest dissociate within 60 min of the resumption of meiosis triggered by major sperm protein signaling (Jud *et al.* 2008). We found that the formation of TIAR-1-containing stress-induced gonad granules depended on translation. These granules dissociated in animals treated with cycloheximide, which is a polysome-stabilizer. Further, puromycin treatment, which destabilizes polysomes, induces the formation of TIAR-1-containing granules in the germline even in the absence of stress (Figure 5).

In mammalian cells, TIA-1 is required for normal stress granule formation (Kedersha *et al.* 1999; Gilks *et al.* 2004). Our results show that, in *C. elegans*, *tiar-1* is required for the formation of stress-induced granules only in the gonad core (Figure 7). Interestingly, even in the absence of *tiar-1*, oocyte granules continued to form. Further, our immunostaining experiments suggest that TIAR-1 and CGH-1 do not exclusively colocalize and that some CGH-1-containing granules form in oocytes in stressful conditions in the absence of TIAR-1. These findings suggest that gonad core and oocytes granules might form using distinct mechanisms. Consistent with this hypothesis, only oocyte RNP granules have been described as utilizing nuclear envelope blebbing and requiring nucleoporins for their formation (Patterson *et al.* 2011). Furthermore, a small proportion of *tiar-1(0)* animals still formed gonad core granules, and neither *tiar-2* nor *tiar-3* significantly influenced the formation of these granules (Table 3). These results suggest the existence of a *tiar*-independent granule formation mechanism. One possibility is that P granule components also play a role in normal gonad granule formation, as has been shown for CGH-1 and CAR-1 on prolonged meiotic arrest granules (Noble *et al.* 2008; Hubstenberger *et al.* 2013, 2015). Another possibility is that other proteins with intrinsically disordered domains induce stress granule formation in the *C. elegans* gonad, just as the MEG (maternal-effect germline defective) proteins promote P-granule assembly in early *C. elegans* embryos (Wang *et al.* 2014), and G3BP, a phosphorylation-dependent endo-ribonuclease that interacts with RasGAP, promotes stress granule assembly in mammalian cells (Tourrière *et al.* 2003).

### tiar-1 protects female germ cells and embryos from heat shock

In *C. elegans*, oocytes grow and obtain their cytoplasm, organelles, and other components via actin-dependent cytoplasmic streaming from the gonad core (Wolke *et al.* 2007). Stress granules form in both the gonad core and oocytes (this work; Boag *et al.* 2008; Jud *et al.* 2008; Noble *et al.* 2008). It has been proposed that stress granules might protect mRNAs from degradation, which would ultimately be introduced into maturing oocytes to support embryogenesis after fertilization (for review, see Schisa 2014). This process might be crucial for embryonic development because embryos rely on maternally contributed mRNAs to start embryogenesis (for review, see Farley and Ryder 2008). Given the relevance of TIA-1/TIAR to mRNA regulation under stressful conditions, we hypothesized that a lack of *tiar-1* might have detrimental effects on germ cells and embryos exposed to stress. After recovery from the heat shock, wild-type germ cells developed into viable embryos but a significant proportion of *tiar-1(0)* germ cells did not (Figure 8B and Figure 9C). Thus, *tiar-1* promotes reproduction under heat stress. Our assay was designed to evaluate the roles of *tiar-1* in developing oocytes under heat shock. Future studies are needed to determine if *tiar-1* protects developing male germ cells from stress as well.

It has been a challenge to separate the function of stress granules from the function of their individual components (for review, see Buchan and Parker 2009). In *C. elegans*, there is evidence that the stress granules formed in oocytes during prolonged meiotic arrest are not detrimental to embryogenesis (Jud *et al.* 2008). In another study, the silencing of *kgb-1* (a member of the JNK subfamily of MAP kinases) led to a low-penetrance lack of oocyte granules (Patterson *et al.* 2011). These oocytes deficient for oocyte granules give rise to embryos that died more often than those with normal numbers of granules (Patterson *et al.* 2011). Nevertheless, the cause of the increase in embryonic lethality is unclear; some oocytes could be affected by the absence of *kgb-1* in processes that are distinct from granule formation. Unexpectedly, our results suggest that gonad core granules might be dispensable for TIAR-1 to protect oocytes from heat shock. We found that wild-type germ cells were able to form viable embryos after heat shock, even in pretreated animals in which large gonad core granules are largely absent (Figure 8B and Figure 9C, Groups II). Further, *tiar-1* mutant animals were similarly sensitive to heat shock irrespective of the temperature at which they were grown. Together, these results are consistent with the idea that large core granules are not essential for the protection of germ cells and embryos from heat stress. Nonetheless, we did observe that some pretreated animals formed small TIAR-1-containing granules in the gonad core (Figure 9B). Further, we observed that some CGH-1-containing granules could form in oocytes after heat shock in the absence of TIAR-1. Thus far, we cannot rule out the possibility that these granules are sufficient to protect germ cells from heat shock.

Interesting findings in mammalian cells suggest that the formation of stress granules is not crucial for mRNA turnover and stabilization (Ohn *et al.* 2008; Bley *et al.* 2014). mRNA turnover from the polysomal to the monosomal fraction under stress is not altered when factors needed for stress-granule formation are silenced (Ohn *et al.* 2008). Similarly, even when stress granules are not formed, the block in translation under stress remains unaffected (Bley *et al.* 2014). A parallel situation occurs with P bodies; even when their formation is disrupted, RNA-mediated gene silencing can still occur, suggesting that dispersed components are competent to perform their function (Eulalio *et al.* 2007).

It is likely that *tiar-1* is needed for the normal response to heat shock in *C. elegans*. Possibly, heat-shocked *tiar-1(0)* mutant oocytes might not be able to cope with heat stress because they sustain cellular damage or are not proficient in damage repair. Using a somatic marker for heat shock, we provide evidence that *tiar-1* mutants are defective in the systemic heat-shock response. The extent to which this systemic defect contributes to the failure of *tiar-1* mutants to form stress-induced granules and protect germ cells from stress is unclear. Alternatively, it might be that the formation of TIAR-1-containing granules contributes in some manner to the robustness of the heat-shock response. Future biochemical studies will be important for delineating the molecular mechanisms by which TIAR-1 protects female germ cells from heat stress in *C. elegans*.

## 

## Supplementary Material

Supplemental Material
