# Supplementary material for: The Stress Granule RNA-Binding Protein TIAR-1 Protects Female Germ Cells from Heat Shock in Caenorhabditis elegans
Source: G3 (Bethesda). 2016 Feb 9;6(4):1031–47. doi: 10.1534/g3.115.026815 (PMC4825639; doi:10.1534/g3.115.026815)
Supplement: Supplemental Material [file supp_6_4_1031__index.html]

The Stress Granule RNA-Binding Protein TIAR-1 Protects Female Germ Cells from Heat Shock in Caenorhabditis elegans — Supplemental Material 

# The Stress Granule RNA-Binding Protein TIAR-1 Protects Female Germ Cells from Heat Shock in *Caenorhabditis elegans*

## Supplemental Material for Huelgas-Morales *et al.*, 2016

**Files in this Data Supplement:**

- Table S1 - Strains used in this study. (.pdf, 30 KB)
- Table S2 - Primers used to produce new *tiar-1* alleles with CRISPR-Cas9 genome editing. (.pdf, 33 KB)
- Table S3 - Infertility of *tiar-1* mutant strains at 25°. (.pdf, 87 KB)
