# Supplementary material for: The Stress Granule RNA-Binding Protein TIAR-1 Protects Female Germ Cells from Heat Shock in Caenorhabditis elegans
Source: G3 (Bethesda). 2016 Feb 9;6(4):1031–47. doi: 10.1534/g3.115.026815 (PMC4825639; doi:10.1534/g3.115.026815)
Supplement: Supplemental Material [file supp_g3.115.026815_TableS1.pdf]

Table S1. Strains used in this study.

Strain	Genotype
N2	Wild type, Bristol isolate
DG1612	*vab-1(dx31)/mIn1 [dpy-10(e128) mIs14]* II; *fog-2(q71)* V
DG3862	*unc-4(e120) vab-1(dx31)* II
DG3883	*tiar-1(tm361) unc-4(e120)* II
DG3922	*tiar-1(tn1545[tiar-1::gfp::tev::s])* II
DG3929	*tiar-1(tn1543)* II
HT1593	*unc-119(ed3)* III
JK987	*tra-2(q276)/mnC1 dpy-10(e128) unc-52(e444)* II
RN054	*tiar-1(tn1545)* II*; fog-2(q71)* V
RN055	*tiar-1(tn1543)* II*; tiar-3(ok144)* X
RN063	*tiar-1(tn1543) tiar-2(tm2923)* II*; tiar-3(ok144)* X
RN064	*tiar-1(tn1543) tiar-2(tm2923)* II
RN065	*tiar-1(tn1543)* II; *gpIs1[Phsp-16-2::GFP]*[a]
RN066	*tiar-1(tn1543)* II; *fog-2(q71)* V
RN067	*tiar-3(ok144)* X
RN068	*tiar-2(tm2923)* II
RN076	*tiar-1(tm361)* II
RN077	*tiar-1(tm361) xmSi02[Ptiar-1::tiar-1::gfp::tiar-1 3utr; Cb-unc-119(+)] II*
TJ375	*gpIs1[Phsp-16-2::GFP]*[a]

[a] The LG for the insertion has not been defined.
