# Supplementary material for: The Stress Granule RNA-Binding Protein TIAR-1 Protects Female Germ Cells from Heat Shock in Caenorhabditis elegans
Source: G3 (Bethesda). 2016 Feb 9;6(4):1031–47. doi: 10.1534/g3.115.026815 (PMC4825639; doi:10.1534/g3.115.026815)
Supplement: Supplemental Material [file supp_g3.115.026815_TableS2.pdf]

Table S2. Primers used to produce new *tiar-1* alleles with CRISPR-Cas9 genome editing.

Name	Sequence	Use in genome editing[a]
001 Nest upstream fow	ggacgctttttggctacttg	
002 Nest upstream rev	accgtttccgagatgaaatc	*tiar-1* template
003 Nest orf+UTR fow	tcccgcactgtttttattgg	from genomic
004 Nest orf+UTR rev	aacctcaacccccagaaatg	DNA
005 Nest downstream fow	ttttgcaatggcacgtattc	
007 Del BlueS rev	ATCGATACCGTCGACCTC	
008 Del tiar upstATG fow	ggtcgacggtatcgatTTAAGAAGACCAGCAGCG	
009 Del tiar upstATG rev	acgaagttatGGCTTAAATGATAGATCCCTG	
010 Del unc-119 fow	atttaagccataacttcgtatagcatacattatacgaagttatCTAGA ATCCTATGCTTGCAC	*tiar-1* deletion:
011 Del unc-119 rev	acgaagttatTTATGCATCATATGAGTAGTCG	Gibson assembly
012 Del tiar-1 downs fow	gatgcataaataacttcgtataatgtatgctatacgaagttatATTGA GTTCGAAAATGTTTATTTCCATTTC	
013 Del tiar-1 downs rev	ctagaactagtggatccCGCCGGTAGGTTTCTAGC	
014 Del BlueS fow	GGATCCACTAGTTCTAGAGC	
015 Ins BlueS rev	ATCGATACCGTCGACCTC	
016 Ins tiar orf fow	tcgaggtcgacggtatcgatATGTCCTTCTTCAACCCAC	
017 Ins tiar orf rev	tctcctttctTTGATGTCCTCCAGAGTTC	
018 Ins gfp fow	aggacatcaaAGAAAGGAGACAGCTGCAG	*tiar-1* GFP
019 Ins gfp rev	atactggagtCTATTTGTATAGTTCATCCATGCC	fusion Gibson
020 Ins tiar 3'utr fow	atacaaatagACTCCAGTATTTTTTAAAAACATTTTA TATG	assembly
021 Ins tiar 3'utr rev	tacgaagttatAAGACTTTACCCTCGCATTTATTATTG	
022 Ins unc-119 fow	gtaaagtcttataacttcgtatagcatacattatacgaagttatCTAG	

	AATCCTATGCTTGCAC	
023 Ins unc-119 rev	tacgaagttatTTATGCATCATATGAGTAGTCG	
024 Ins tiar downs fow	TGATGCATAAataacttcgtatagcatacattatacgaagttatat tgagttcgaaaatgtttatttc	
025 Ins tiar downs rev	gctctagaactagtggatccCGCCGGTAGGTTTCTAGC	
026 Ins BlueS fow	GGATCCACTAGTTCTAGAGC	
048 sgRNA 2 tiar-1 fow	gccgtactcGTTTTAGAGCTAGAAATAGCAAG	sgRNA *tiar-1*
049 sgRNA 2 tiar-1 rev	tgtgagtttcCAAGACATCTCGCAATAGG	

[a] Details of strain constructions are available upon request.
