# Supplementary material for: The Stress Granule RNA-Binding Protein TIAR-1 Protects Female Germ Cells from Heat Shock in Caenorhabditis elegans
Source: G3 (Bethesda). 2016 Feb 9;6(4):1031–47. doi: 10.1534/g3.115.026815 (PMC4825639; doi:10.1534/g3.115.026815)
Supplement: Supplemental Material [file supp_g3.115.026815_TableS3.pdf]

Table S3. Infertility of *tiar-1* mutant strains at 25º

Genotype	% Infertility
tiar-1(tm361)[a]	76 (n=71)
tiar-1(tm361) unc-4(e120)[b]	21 (n=128)
tiar-1(tn1543)[c]	21 (n=105)
tiar-1(tn1543) tiar-2 (tm2923);tiar-3(ok144)	25 (n=93)

Strains of the indicated genotype were grown at 25ºC. Two day-old adult animals were mounted and observed under the microscope. A hermaphrodite was considered as “Infertile” if it did not contain a single embryo at the observed time point.

[a] The infertility of this strain was quantified before backcrossing it by following recombination events with the strain *unc-4(e120) vab-1(dx31)*.

[b] 36% of *tiar-1(tm361) unc-4(e120)* hermaphrodites grown continually at 25°C exhibited sterility (n=239). Animals were scored as sterile if they produced no viable progeny.

[c] 30% of *tiar-1(tn1543)* hermaphrodites grown continually at 25°C exhibited sterility (n=300). Animals were scored as sterile if they produced no viable progeny.
